# Exploiting noise as a resource for computation and learning in spiking neural networks

**DOI:** 10.1016/j.patter.2023.100831

**Published:** 2023-09-04

**Authors:** Gehua Ma, Rui Yan, Huajin Tang

**Affiliations:** 1 College of Computer Science and Technology, Zhejiang University, Hangzhou, PRC; 2 College of Computer Science and Technology, Zhejiang University of Technology, Hangzhou, PRC; 3 State Key Lab of Brain-Machine Intelligence, Zhejiang University, Hangzhou, PRC

**Keywords:** spiking neural network, noisy spiking neural network, surrogate gradient, noise-driven learning, neuromorphic intelligence, neural coding, probabilistic graphical model, dynamic system

## Abstract

Networks of spiking neurons underpin the extraordinary information-processing capabilities of the brain and have become pillar models in neuromorphic artificial intelligence. Despite extensive research on spiking neural networks (SNNs), most studies are established on deterministic models, overlooking the inherent non-deterministic, noisy nature of neural computations. This study introduces the noisy SNN (NSNN) and the noise-driven learning (NDL) rule by incorporating noisy neuronal dynamics to exploit the computational advantages of noisy neural processing. The NSNN provides a theoretical framework that yields scalable, flexible, and reliable computation and learning. We demonstrate that this framework leads to spiking neural models with competitive performance, improved robustness against challenging perturbations compared with deterministic SNNs, and better reproducing probabilistic computation in neural coding. Generally, this study offers a powerful and easy-to-use tool for machine learning, neuromorphic intelligence practitioners, and computational neuroscience researchers.

## Highlights

• An SNN framework that yields scalable, flexible, and robust computation
• Scalable spiking models that incorporate noisy neuronal dynamics
• We reveal that unreliable neural substrates yield reliable computation and learning
• NSNN facilitates the efficient employment of various neuromorphic hardware designs

## The bigger picture

Spiking neural networks (SNNs) are a type of computational model designed to mimic aspects of biological neuronal networks and are seen as a promising bridge between artificial and biological intelligence. A biological brain can perform effective computations in the presence of internal or external noise, but until now, most SNN models were deterministic in nature. To understand how noisy biological systems can function reliably and effectively and to develop bio-inspired artificial intelligence (AI), it is vital to build large-scale computational models with noisy components. This work introduces such a noisy SNN model, which is then demonstrated to have various advantages over deterministic models. SNN models that incorporate noise, like this one, may provide a promising avenue to advance research on neuromorphic intelligence and for adapting artificial neural networks to specialized neuromorphic hardware.

## Teaser

This work presents a theoretical framework termed noisy spiking neural network (NSNN) to exploit the computational advantages originating from non-deterministic, noisy neural dynamics in the brain. Incorporating neuronal noise using NSNN improves the performance of spiking neural networks, demonstrates reliable and effective learning, and better reproduces the variability observed in biological neural computation. Because NSNN subsumes traditional deterministic spiking models, this model is advantageous for enabling more flexible and robust computation on neuromorphic hardware with inherent unreliabilities.

## Introduction

Spiking neural networks (SNNs) are seen as a promising way to bridge the gap between artificial and biological neural networks. They have been widely used as computational models in neuroscience research.^1^^,^^2^ Benefiting from the recent progress in deep learning,^3^^,^^4^^,^^5^ SNNs have also achieved remarkable advantages in various applications such as computer vision and robotics.^6^^,^^7^^,^^8^^,^^9^^,^^10^^,^^11^^,^^12^^,^^13^^,^^14^ In general, the majority of spiking neural models are established by deterministic SNNs (DSNNs), which ignore the inherent randomness of spiking neurons. Spiking neurons with noise-perturbed dynamics are considered more biologically realistic, as the ion channel fluctuations and synaptic transmission randomness can cause noisy sub-threshold membrane voltages.^15^^,^^16^^,^^17^^,^^18^^,^^19^^,^^20^^,^^21^^,^^22^ Furthermore, the internal noise incurs a potential benefit in generalization performance by promoting a more fault-tolerant representation space^23^^,^^24^^,^^25^ and preventing overfitting.^26^ However, a generic and flexible approach is required to fully exploit the noisy spiking neural models and comprehend the role of noise in networks of spiking neurons.

Previous literature^27^^,^^28^ has investigated spiking neurons with stochastic activity by including a noise term in the differential equation of the membrane voltage. Such a noise term is typically modeled as white or colored noise derived in the stochastic differential equation with a diffusion process.^29^^,^^30^ While these approaches introduced detailed spiking models with noise-perturbed neuronal dynamics, they do not attempt to build a generic method on the network level. Some studies^31^^,^^32^^,^^33^^,^^34^ have presented small networks of noisy spiking neurons that can perform probabilistic inference. Nevertheless, these methods are difficult to incorporate into arbitrary network architectures due to the lack of an effective learning method. A recent study^35^ introduced a generalized linear model variant of the deterministic spike response model, but their method does not scale to deep SNNs of interest here. In deep SNNs, an *ad hoc* solution called surrogate gradient learning^36^^,^^37^^,^^38^ (SGL; pseudo-derivative^39^) has become the most widely used to solve the discontinuity when performing back-propagation.^40^ While the surrogate gradient methods have proven highly effective,^41^ they lack a theoretical foundation and a rational explanation.^42^ In contrast, neuroscience-informed learning methods, like STDP,^43^^,^^44^^,^^45^ are theoretically grounded and have shown promise, but struggle to work well in large networks and for complex tasks. It is therefore necessary to develop an effective and scalable learning method like SGL while retaining insights regarding learning mechanisms like STDP.

The previous lack of a general computation and learning co-design of noisy spiking neural models has impeded their use. This prevents us from fully exploiting the performance of noisy SNNs as machine-learning models and exploring their potential as computational tools for neuroscience. Thus, this article aims to provide a generic and flexible integration of deep SNNs and noisy spiking neural models. In this way, we can make the spiking neural model biologically more realistic and realize potential performance gains while being able to directly benefit from the engineering advances emerging from the rapidly growing deep learning field. Moreover, in terms of theoretical value, this also demonstrates how noise may act as a resource for computation and learning in general networks of spiking neurons.^46^

Here, we show a noisy SNN (NSNN) model using a noise-driven learning (NDL) paradigm. The approach shown here provides a generic and flexible integration of noisy spiking neural models and deep SNNs. In addition, NDL subsumes SGL and provides an insightful explanation for the latter. By incorporating various SNN architectures and algorithms, we demonstrate the effectiveness of NSNNs. Also, NSNNs lead to significantly improved robustness when facing challenging perturbations, such as adversarial attacks. In addition, through NSNN-based neural coding analyses, we show the potential of the NSNN model as a useful computational tool for neuroscientific research.

## Results

### Noisy spiking neural network and noise-driven learning

**Figure 1:**
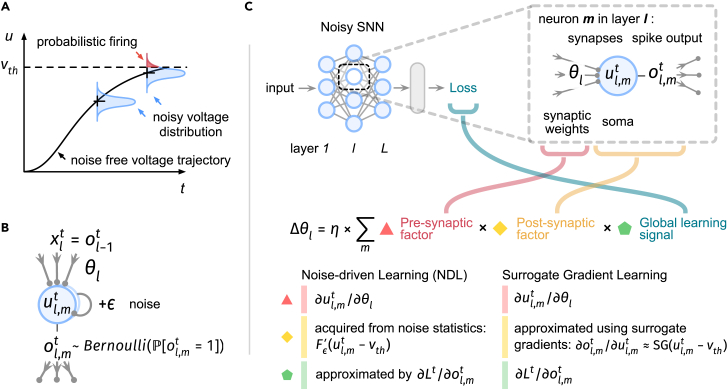
Schemes for NSNN and NDL (A) Introducing noisy neuronal dynamics yields a probabilistic firing mechanism, where the firing probability is given by the membrane noise cumulative distribution function, indicated by the shaded red part under the noisy voltage distribution. Here, *u* denotes membrane voltage and $v_{\text{th}}$ denotes firing threshold. (B) Computation flow in a noisy LIF neuron l,m with membrane voltage $u_{l,m}^{t}$, input $o_{l-1}^{t}$, and output $o_{l,m}^{t}$. (C) Illustration of the computation for updating synaptic weights $\theta_{l}$ of layer *l* using NDL and SGL. $F_{\epsilon }$ denotes the cumulative distribution function of noise ϵ, and η denotes learning rate.

We consider a noisy leaky integrate-and-fire (LIF) spiking neuron model (see the experimental procedures under “method details”) in this article, following previous literature that uses diffusive approximation.^29^^,^^30^^,^^47^ It considers a discrete sub-threshold equation of the form:

(Equation 1) $$\text{Noisy}\;\text{LIF}\;\text{sub}\text{-}\text{threshold}\;\text{dynamic}:u^{t}=\tau u^{t-1}+\phi_{\theta }\left(x^{t}\right)+\epsilon \text{,}$$

where *u* denotes membrane potential, $x^{t}$ denotes input at time *t*, τ is the membrane time constant, and $\phi_{\theta }$ is a parameterized input transform. The noise ϵ is drawn from a zero-mean Gaussian distribution. Introducing internal noise induces a probabilistic firing mechanism (illustrated in Figure 1A), where the difference $u-v_{\text{th}}$ governs the firing probability^20^^,^^29^^,^^30^:

(Equation 2) $$\text{Noisy}\;\text{LIF}\;\text{probabilistic}\;\text{firing}:o^{t}∼\text{Bernoulli}\left(P\left[o^{t}=1\right]\right)\text{,}$$

where $o^{t}$ is the spike state, $P\left[o^{t}=1\right]=F_{\epsilon }\left(u^{t}-v_{\text{th}}\right)$, $F_{\epsilon }$ is the cumulative distribution function of the noise, and $v_{\text{th}}$ is the firing threshold.

By employing noisy LIF neurons, the NSNN presents a general form of SNNs. For instance, if the noise variance Var[ϵ] approaches zero, the firing probability function $F_{\epsilon }$ converges to the Heaviside step function, and the noisy LIF model, therefore, subsumes the deterministic LIF case. This suggests that conventional DSNNs can be viewed as a special type of NSNN. Further, by considering logistic membrane noise, the noisy LIF neuron covers the sigmoidal neuron model.^46^

We then provide a well-defined solution of synaptic optimization via gradient descent for networks of spiking neurons. As noisy neurons code for binary variables, we may represent NSNNs using the Bayesian network,^48^^,^^49^ a probabilistic graphical model representing a set of variables and their conditional dependencies by a directed graph. Leveraging the Bayesian network formulation, we can succinctly represent the spike states in NSNNs. This enables us to convert the gradient computation during synaptic optimization into a gradient estimation in a probabilistic model, thereby circumventing the problematic firing function derivative. The resulting NDL rule (see the experimental procedures under “method details”) is illustrated in Figure 1C. In particular, the concise form of NDL makes it easy to combine with other online^39^^,^^50^ or local learning^51^ strategies to utilize computational resources more efficiently and handle more diverse tasks.

**Figure 2:**
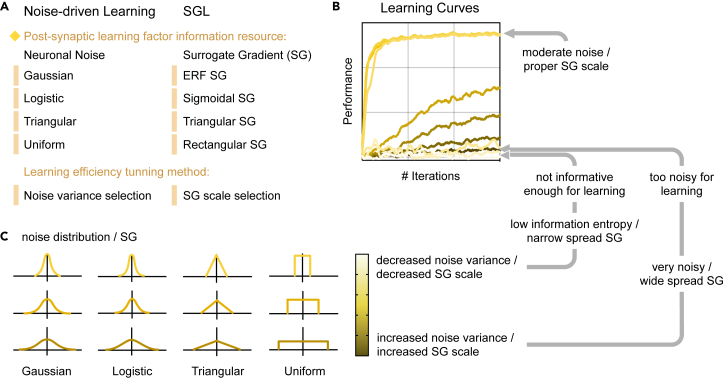
NDL provides a principled backend for SGL (A) The correspondence between various types of neuronal noise and surrogate gradient functions. When using surrogate gradients, we obtain statistical information from the noisy membrane voltage dynamics to form a post-synaptic learning factor. (B and C) The effects of adjusting noise variance or surrogate gradient scale on learning efficiency.

One noteworthy feature of NDL is that it provides a principled rationale for SGL. By leveraging the three-factor learning rule framework,^52^^,^^53^ we show the mathematical relationship presented in Figure 1C, indicating that SGL can be regarded as a special type of NDL. Although the surrogate gradients^7^^,^^36^^,^^37^^,^^41^ have been commonly associated with the straight-through estimator^54^^,^^55^^,^^56^^,^^57^ in binarized networks, this association is not justified from the perspective of spiking neurons. In this sense, SGL appears to be an *ad hoc* solution rather than a theoretically sound learning method.^36^^,^^42^ NDL reveals the essence of the surrogate gradient, which is to obtain the post-synaptic learning factor from the neuron membrane noise statistics. Figure 2A illustrates the relationship between noise-variance selection in NDL and surrogate-gradient-scale selection in SGL. When the noise variance is small, the probabilistic inference forward of an NSNN can be approximated by the deterministic forward pass in a DSNN. Therefore, NDL derived within the NSNN framework subsumes SGL in conventional DSNNs. This also provides a random noise explanation for adjusting the scale (shape) of the surrogate gradient functions in SGL. Figures 2B and 2C visualize the impact of this variance-selection or scale-selection process on the learning effect of SNNs, demonstrating that adjusting the scale of the surrogate gradient functions in SGL can be viewed as selecting noises with different variances in NDL.

### NSNN leads to high-performance spiking neural models

To verify the effectiveness and compatibility of NSNNs, we conducted experiments on multiple recognition benchmark datasets using various combinations of SNN architectures and algorithms. Recognition datasets we considered include static-image datasets, including CIFAR-10 and CIFAR-100,^58^ and event-stream datasets collected using DVS cameras (silicon retina), including DVS-CIFAR^59^ and DVS-Gesture.^60^ More experimental details are presented in the experimental procedures under “experimental details.”

We analyzed the accuracy of the models on these datasets, and we mainly focus on comparison with SNN models here. As shown in Table 1, the results on CIFAR-10 and CIFAR-100 demonstrate the effectiveness of NSNNs on static image recognition tasks. For instance, on CIFAR-10, the NSNN model (with STBP, CIFARNet) achieved an accuracy of 0.9390 for two simulation time steps, while its deterministic counterpart reported 0.9188. Our results indicate that NSNNs work well with various SNN architectures and algorithms, demonstrating great compatibility. Therefore, NSNNs can benefit from more efficient SNN algorithms or architectures. For example, when using the ResNet-18 architecture, the NSNN model using the more efficient TET algorithm performs significantly better than the NSNN model using STBP (Table 1; CIFAR-10, CIFAR-100). NSNNs also demonstrate effectiveness and compatibility on event-stream-data-recognition tasks (Table 1; DVS-CIFAR, DVS-Gesture) and outperformed their DSNN counterparts. In particular, on DVS-CIFAR data, the NSNN model with STBP and ResNet-19 significantly outperforms its DSNN counterpart, and performance improvements in other combinations are also evident. Due to the limited number of samples, DSNNs often experience severe overfitting when working with DVS-CIFAR and DVS-Gesture data. However, in NSNNs, internal noise improves the model’s generalization ability, resulting in better performance than their deterministic counterparts. These generalization improvements brought about by internal noise in NSNNs align with previous research results in artificial neural networks (ANNs).^24^^,^^25^^,^^26^

**Table 1:** Evaluation results on CIFAR-10, CIFAR-100, DVS-CIFAR, and DVS-Gesture datasets

NSNN	SNN algorithm	SNN architecture	Accuracy	Accuracy
CIFAR-10				

–	–	–	time step = 2	time step = 4
–	STCA^111^	CIFARNet	91.23 (time step = 12)	–
–	STBP-tdBN^110^	ResNet-19	92.34	92.92
–	STBP^7^	ResNet-18	93.18 ± 0.07	93.93 ± 0.11
Yes	STBP	ResNet-18	92.87 ± 0.04	93.77 ± 0.12
–	STBP	CIFARNet	91.88 ± 0.09	92.79 ± 0.14
Yes	STBP	CIFARNet	93.90 ± 0.12	94.30 ± 0.08
–	TET^9^	ResNet-18	93.62 ± 0.02	94.09 ± 0.20
Yes	TET	ResNet-18	93.12 ± 0.07	94.14 ± 0.05

CIFAR-100				

–	–	–	time step = 2	time step = 4
–	TET	ResNet-19	72.87 ± 0.10	74.47 ± 0.15
–	STBP-tdBN	ResNet-19	69.41 ± 0.08	70.86 ± 0.22
–	STBP	ResNet-18	70.15 ± 0.14	70.88 ± 0.19
Yes	STBP	ResNet-18	69.57 ± 0.09	71.16 ± 0.40
–	STBP	CIFARNet	72.25 ± 0.08	72.94 ± 0.21
Yes	STBP	CIFARNet	73.36 ± 0.14	74.17 ± 0.28
–	TET	ResNet-18	71.72 ± 0.13	74.01 ± 0.43
Yes	TET	ResNet-18	71.34 ± 0.09	73.33 ± 0.03

DVS-CIFAR				

–	–	–	time step = 10	–
–	Fang et al.^12^	Wide-7B-Net	74.40 (time step = 16)	–
–	Wu et al.^112^	LIAFNet	71.70	–
–	STBP	ResNet-19	71.74 ± 0.92	–
Yes	STBP	ResNet-19	74.30 ± 0.61	–
–	STBP-tdBN	VGGSNN	75.51 ± 0.49	–
Yes	STBP-tdBN	VGGSNN	76.97 ± 0.10	–
–	TET	VGGSNN	78.26 ± 0.17	–
Yes	TET	VGGSNN	79.52 ± 0.38	–

DVS-Gesture				

–	–	–	time step = 16	–
–	Fang et al.^12^	7B-Net	97.92	–
–	STBP-tdBN	ResNet-17	96.87 (timestep = 40)	–
–	STBP	7B-Net	95.84 ± 0.27	–
Yes	STBP	7B-Net	96.88 ± 0.28	–

Data are represented as the mean ± SD.

### NSNN leads to improved robustness against challenging perturbations

Robustness is essential for the information processing of spiking neural models to prevent external perturbations and interferences in real-world environments. From an application standpoint, robustness ensures reliable performance when facing corrupted input (possibly caused by errors in data collection and processing) and perturbed internal information flow (possibly caused by communication abnormalities between units). In terms of building biologically realistic computational neural circuits, robust spiking neural models align more closely with the noisy yet resilient characteristics of biological circuits.^19^^,^^61^^,^^62^

Next, we demonstrate the improvement in robustness achieved by using NSNNs. To this end, we conducted perturbed recognition experiments on CIFAR-10, CIFAR-100, and DVS-CIFAR data (see the experimental procedures under “experimental details”). We evaluated the performance of DSNNs and NSNNs by measuring their accuracy and loss values under various types and intensities of perturbations. The models were trained as described in the previous recognition experiments and tested using input-level or spike-state-level perturbations.

We considered several challenging input-level perturbations. For static-image data CIFAR-10 and CIFAR-100, we used challenging adversarial attacks to construct corrupted inputs. This work leveraged two adversarial attacks: the fast gradient sign method (FGSM) and the direct optimization method (DO). For event-stream data DVS-CIFAR, we used the EventDrop^63^ perturbation, whose basic idea is randomly dropping a proportion of events with a probability of ρ. We also considered directly perturbing all spike states (firing states of spiking neurons) in SNNs to directly mimic the spike-train variability in biological neural circuits. The intensity of the perturbation is controlled by parameter β. Details are presented in the experimental procedures.

**Figure 3:**
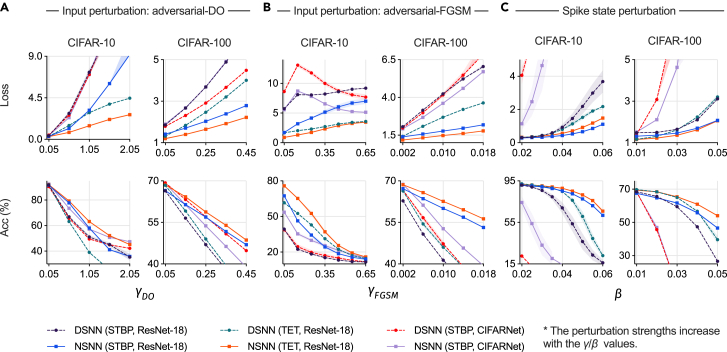
Results of perturbed recognition experiments on CIFAR-10 and CIFAR-100 datasets (A and B) Loss and accuracy results under input-level adversarial attacks (DO method, FGSM). (C) Loss and accuracy results under spike-state-level (firing state of spiking neurons) perturbations.

Figure 3 shows that using NSNNs significantly improved robustness for static-data-recognition tasks (CIFAR-10, CIFAR-100). When facing challenging adversarial attacks and hidden-state perturbations, NSNN models consistently outperformed their DSNN counterparts. For example, in the CIFAR-100 FGSM adversarial attack experiment (Figure 3B), NSNNs demonstrated good resilience, whereas the reliability of DSNNs degraded radically as the perturbation intensity increased. Similarly, when facing direct interference with neuron spike states (Figure 3C), NSNNs exhibited superior robustness compared with their deterministic counterparts. The perturbed experiments on DVS-CIFAR data also showed that the NSNN model achieved better robustness than DSNNs (Tables 2 and 3). When facing input-level EventDrop perturbations, NSNNs achieved lower loss and higher accuracy than their deterministic counterparts in most cases (Table 2). Similarly, as shown in Table 3, when facing hidden state perturbations, NSNNs demonstrated good resilience. Their superiority becomes apparent as the perturbation (spike state) level increases. For example, at a perturbation level of β=0.01 (with STBP and ResNet-19), the accuracy of NSNNs was 4.5% higher than that of DSNNs. When the perturbation level increased to 0.04, the accuracy lead of NSNNs over DSNNs reached 46.3%. We provide a theoretical analysis of internal noise and model stability in the following text (see the experimental procedures under method details under “theoretical analyses on internal noise and stability”).

**Table 2:** Evaluation results under EventDrop input-level perturbation on DVS-CIFAR data

Algorithm and architecture	NSNN	ρ = 0.05	ρ = 0.25	ρ = 0.45	ρ = 0.65
**Loss**					

STBP and ResNet-19	–	2.27 ± 0.16	6.89 ± 2.05	8.60 ± 1.55	9.06 ± 1.08
	yes	1.80 ± 0.09	5.84 ± 0.76	7.65 ± 1.21	8.55 ± 1.40
tdBN and VGGSNN	–	2.24 ± 0.14	6.31 ± 0.74	8.25 ± 1.49	9.49 ± 1.61
	yes	1.90 ± 0.06	6.91 ± 0.16	8.19 ± 0.82	8.66 ± 1.35
TET and VGGSNN	–	1.21 ± 0.01	2.88 ± 0.31	3.44 ± 0.31	4.13 ± 0.57
	yes	1.03 ± 0.05	2.55 ± 0.25	4.02 ± 0.29	4.15 ± 0.18

**Accuracy (%)**					

STBP and ResNet-19	–	60.09 ± 2.46	17.68 ± 5.72	13.21 ± 1.31	12.42 ± 0.29
	yes	65.66 ± 1.80	25.31 ± 5.72	16.36 ± 3.23	13.32 ± 0.61
tdBN and VGGSNN	–	64.98 ± 1.63	26.64 ± 3.32	18.41 ± 2.43	13.74 ± 1.00
	yes	70.28 ± 1.36	30.14 ± 0.99	22.55 ± 2.07	18.78 ± 1.97
TET and VGGSNN	–	67.86 ± 0.43	29.26 ± 4.34	20.76 ± 2.45	15.70 ± 2.80
	yes	71.67 ± 1.20	29.14 ± 1.16	21.34 ± 0.73	14.73 ± 0.29

A larger ρ denotes stronger perturbation. Data are represented as the mean ± SD.

**Table 3:** Evaluation results under spike-state-level (firing state of spiking neurons) perturbation on DVS-CIFAR data

Algorithm and architecture	NSNN	β = 0.01	β = 0.02	β = 0.03	β = 0.04
**Loss**					

STBP and ResNet-19	–	1.43 ± 0.04	1.72 ± 0.06	2.44 ± 0.03	3.43 ± 0.25
	yes	1.23 ± 0.04	1.30 ± 0.03	1.41 ± 0.13	1.74 ± 0.32
tdBN and VGGSNN	–	1.29 ± 0.05	1.30 ± 0.08	1.49 ± 0.18	1.88 ± 0.29
	yes	1.25 ± 0.01	1.19 ± 0.02	1.22 ± 0.06	1.50 ± 0.13
TET and VGGSNN	–	0.82 ± 0.03	0.91 ± 0.06	1.08 ± 0.08	1.37 ± 0.06
	yes	0.75 ± 0.01	0.80 ± 0.01	0.94 ± 0.06	1.25 ± 0.14

**Accuracy (%)**					

STBP and ResNet-19	–	69.73 ± 0.88	63.91 ± 1.37	53.60 ± 1.39	40.32 ± 1.91
	yes	72.88 ± 0.69	70.44 ± 0.42	67.27 ± 2.66	58.99 ± 6.80
tdBN and VGGSNN	–	74.09 ± 0.66	70.61 ± 1.37	63.19 ± 2.89	50.74 ± 3.32
	yes	76.16 ± 0.13	73.38 ± 0.55	68.77 ± 0.78	55.64 ± 1.87
TET and VGGSNN	–	76.41 ± 0.92	72.60 ± 1.11	66.46 ± 1.93	56.06 ± 1.05
	yes	78.28 ± 0.27	76.32 ± 1.01	71.54 ± 1.07	62.48 ± 0.52

A larger β denotes stronger perturbation. Data are represented as the mean ± SD.

### NSNN demonstrates a promising tool for neural coding research

**Figure 4:**
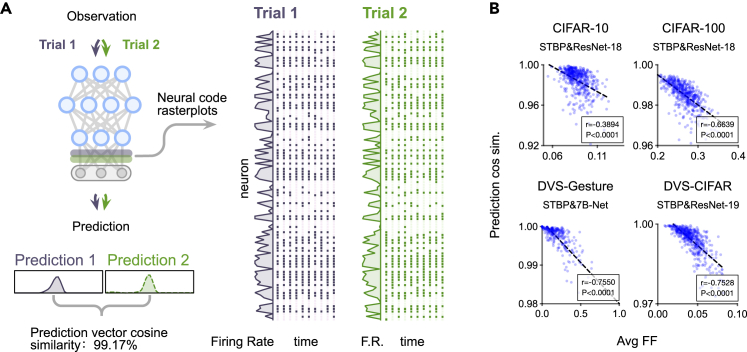
NSNN-based coding analyses (A) NSNNs exhibit neural code-level variability and decision-level (prediction-level) reliability. We visualize the prediction distributions, spike rates, and raster plots of the final spiking layer outputs of two repeated trials obtained using DSV-CIFAR data. (B) Relationship between the average Fano factor and prediction cosine similarity. Each dot represents a sample used for computing the Pearson correlation. The dashed line is a linear approximation.

Although they capture the spike-based paradigm in neural circuits, conventional DSNNs fail to account for the reliability and variability in neural spike trains,^64^^,^^65^ which limits their application as computational models in neural coding research. By contrast, NSNNs can faithfully recover prediction reliability and neural spike train variability, as shown in Figure 4A. The NSNN, therefore, can be a useful tool for investigating neural coding, where, to provide information about dynamic sensory cues, the patterns of action potentials in spiking neurons must be variable.^66^

We first show how the NSNN can be used for neural coding analysis in spiking neural models. In particular, we leverage the NSNN model to provide empirical evidence for a rate coding strategy. Specifically, the internal randomness of NSNNs leads to slightly different neural codes (spike output of the final spiking layer) and predictions between trials given the same input (Figure 4A). This allows for analyzing the Pearson correlation between the variation in the firing rate of the neural code and the stability (reliability) of the final prediction in NSNNs. Since we consider a recognition task here, we use the final spiking neuron layer output, which contains more semantically informative data, as the neural code. We use the Fano factor (FF) to numerically measure the firing rate variation in neural code^67^ and the cosine similarity of prediction vectors to measure prediction stability (see also the experimental procedures under “experimental details”). A larger FF indicates greater firing rate variation between trials, while high prediction cosine similarity corresponds to more stable predictive output. We find significant negative correlations between variation in firing rate and stability of prediction in learned NSNNs (Figure 4B). These results are robust to various combinations of SNN architectures and algorithms, suggesting that these NSNNs learn a primary rate coding-based policy. This makes sense, as membrane noise injection introduces uncertainty into the firing process, reducing the reliability of precise spiking time-based coding. As the same firing rate (represented as firing count in simulation steps here) can correspond to different spike trains, rate-based coding can improve model robustness by constructing a representation space with better fault tolerance.

**Figure 5:**
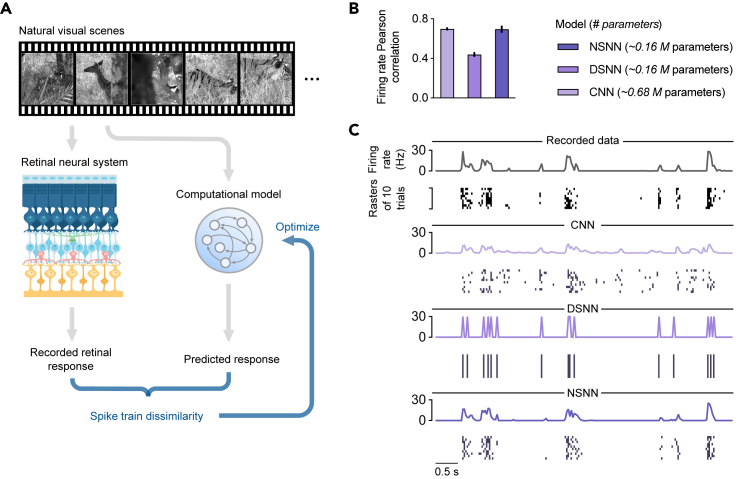
Neural activity fitting experiments (A) Graphical illustration of the simple neural activity fitting experiment: spiking neural models were optimized to produce spike outputs close to the recorded neural activities. (B) The average firing rate Pearson correlation coefficients of different fitting models. Higher values indicate better predictions. (C) Spikes and firing rates of the recorded responses of a representative neuron and the predictions of three models (CNN, DSNN, NSNN) to a natural visual scene clip.

Spiking neural models are widely used as computational tools for neural circuits. One effective way to validate these approaches is to simulate the neural processing process on a computer and verify through quantitative testing whether they can perform the tasks completed by some specific biological neural systems and produce similar results. Therefore, we performed a neural activity fitting task (see also the experimental procedures under “experimental details”) using retinal neural response to natural visual scenes^68^ (Figure 5A) to highlight the advantages of NSNN as a computational model. In particular, we constructed DSNN and NSNN models with the same structure for this neural activity fitting task. These spiking neural models took the visual scenes as input and the recorded retinal responses as target outputs and were optimized to produce spike trains similar to recorded neural activity. To compare these spiking neural models with the current state of the art, we also considered a competitive convolutional neural network (CNN) model^69^^,^^70^ as the baseline. We evaluated the Pearson correlation coefficient between the recorded and the predicted neural activity to compare the performances of these models numerically. To illustrate that the DSNN model failed to account for variability in the neural activity of interest, we show in Figure 5C the spike rasters and firing rates of a representative neuron. As presented in Figure 5C, the DSNN model could not reproduce the variable spike patterns and failed to accurately fit real firing rate curves. In contrast, we observed a significant improvement using the NSNN (Figure 5B). The NSNN model performs well in fitting both spike activity and firing rate. In addition, the NSNN model can demonstrate competitive performance with fewer model parameters than the CNN model. These results demonstrate that applying performance optimization to a biologically appropriate NSNN model makes it possible to construct quantitative predictive models of neural coding.^71^

## Discussion

In this study, we reported noisy spiking neural models and NDL by exploiting neuronal noise as a resource for computation and learning in networks of spiking neurons. We introduced a membrane noise term into the deterministic spiking neuron model,^27^^,^^30^^,^^72^ formulated the networks of these noisy spiking neurons, and derived the NDL method to perform synaptic optimization. Our results on multiple datasets indicate that NSNNs exhibit competitive performance and improved generalization ability. Further perturbed tests show that NSNNs demonstrate improved robustness against various perturbations, including challenging adversarial attacks, thereby providing empirical support for the internal noise-model stability analysis. In addition, NSNNs can easily integrate with various SNN algorithms and network architectures, allowing our methodology to be trivially generalized to a wide range of fields that require the presence of stochasticity. Finally, our exposition on coding strategy analysis and the construction of predictive coding models demonstrates the promising potential of NSNNs as an useful tool for neural coding research. Furthermore, since the general framework presented in this study subsumes traditional deterministic spiking models, our method is expected to enable more flexible and robust computation on neuromorphic hardware with inherent unreliabilities.

### Advancing spiking neural network research

This work presents a novel approach for constructing and training SNNs. The NSNN framework enables the flexible integration of internal noise with different distributions and variances into SNN models. In machine learning, introducing internal noise typically offers performance benefits, such as preventing overfitting and enhancing model robustness. As such, NSNNs can be utilized to obtain better performance models. The computation and learning form of the NSNN incorporated with NDL is concise and straightforward, making it easy to implement in larger network architectures using simple module replacement based on the DSNN implementation. Here we demonstrate that the combination of NSNN and NDL can be well incorporated into representative network architectures and algorithms. Going forward, NSNNs can easily be integrated with larger models, like spiking transformers.^73^^,^^74^ Although we currently assign credits along the temporal dimension using back-propagation through time in experiments, NDL is compatible with potential online or local learning methods. Further research into the noisy spiking neural models may provide insights for designing local, online learning mechanisms^39^^,^^50^^,^^51^ and combining them with NDL to form more biophysically plausible SNN learning methods. As such, NSNN presents a promising avenue for advancing SNNs and their learning methods.

### Incorporation with neuromorphic hardware

As a crucial component of neuromorphic intelligence research,^14^ SNNs are expected to achieve recognition, adaptation, behavior, and learning at low power consumption through integration with neuromorphic hardware.^75^^,^^76^^,^^77^ The NSNN and NDL approach introduced in this article will benefit hardware implementation of spiking neural models on analog, digital, or mixed^78^ platforms. Neuromorphic hardware such as TrueNorth^79^ and Intel Loihi^80^ incorporates built-in pseudo-random number generators or random noise parameters to simulate the unreliability in computation. This enables the implementation of noisy spiking neural models on neuromorphic hardware and extends the computational power of noisy spiking neural models by utilizing these built-in random sources for practical applications. Furthermore, due to the adoption of noisy neuronal dynamics, NSNNs have considerable robustness (demonstrated by previous experiments) against internal disturbances. Therefore, NSNNs are expected to exhibit more stable performance in neuromorphic hardware systems that inherently have noise,^81^^,^^82^ such as electronic disturbances, input perturbations, and communication errors. This allows for more efficient and effective employment of various neuromorphic hardware developments in real-world scenarios. Hence, this work contributes a fundamental theoretical foundation for the future development of neuromorphic computing.

### Noise in biological neural networks

Noise in biological neural networks originates from diverse sources, including voltage or ligand-gated ion channels, the fusion of synaptic vesicles, and the diffusion of signaling molecules to receptors.^19^ Channel noise can also affect membrane potential, spike initiation, and spike propagation in small axons and cell bodies.^83^ In addition, synapses exhibit randomness in the number of transmitter molecules released in a vesicle and in the diffusion processes of molecules.^19^ Early neuroscientists recognized that intrinsic noise in brain activity could confer benefits by randomizing neuronal dynamics, leading to advantages in creativity, probabilistic decision-making, unpredictability, and allocation to discrete categories.^67^ Modern brain signal acquisition and imaging techniques such as EEG, iEEG, and fMRI allow us to explore the role of noise as a computing element in brain networks by pinpointing the locations of different cognitive processes. For instance, a recent study^84^ used iEEG measurement to demonstrate that resting-state cortical noise may influence visual recognition ability. This suggests that noise is not merely a concomitant of neural processing,^67^ but can play a unique and important role in brain networks. By utilizing the NSNN framework in conjunction with brain imaging and electrophysiological signal acquisition techniques, we can establish a tractable computational link between neuronal activity variability and probabilistic behavior. This could further improve our understanding of the role of noise in neural processing and contribute to the comprehension of the mechanisms underlying memory and decision-making in the brain.

### Implications for neuroscientific research

Spiking neural models are popular in neuroscience research for their biologically realistic spike-based computation paradigm. However, conventional deterministic spiking neural models cannot account for the variability in neural spike trains.^66^ Recent neuroscience research^84^ suggests resting-state cortical noise as a possible neurophysiological trait that limits recognition capacity, implying that our brain is non-deterministic. To understand and simulate cognitive functions such as memory, recognition, attention, and decision-making in the noisy brain, we are dealing with large-scale, sophisticated computational systems. Modeling these systems requires more complex neural networks composed of a large number of neurons. Therefore, research into the scalable noisy spiking neural model, which aims to provide a useful tool at a computational level, has practical benefits for computational neuroscience. NSNNs are expected to be employed for neuron type,^2^^,^^85^ neural system identification,^86^^,^^87^ and constructing predictive counterparts of neural circuits.^88^^,^^89^ In the experiments shown in this article, we mainly focus on the potential application of NSNNs in neural coding. This is a popular research topic in neuroscience and other fields such as computer vision, neuromorphic computing, neural prosthesis, and brain-computer interface. Our results indicate that NSNNs are able to recover reliability and variability in neural circuits. Notably, NSNNs achieved competitive performance with fewer parameters than conventional ANN models in fitting neural responses to natural visual scenes. As such, NSNN provides a promising tool for building computational accounts for various sensory neural circuits and will enable richer models of complex neural computations in the brain.

## Experimental procedures

### Resource availability

#### Lead contact

Further information and requests for resources should be directed to and will be fulfilled by the lead contact, Prof. Huajin Tang (htang@zju.edu.cn).

#### Materials availability

This study did not generate new unique reagents.

### Method details

#### Notations

We use x,u,o to represent input, membrane potential, and spike output. Also, $x_{l,m}^{t},u_{l,m}^{t},o_{l,m}^{t}$ denote variables of neuron *m* in layer *l* (whose dimension is $\dim\left(l\right)=\dim\left(x_{l}\right)$) at time *t*, where l∈[1,L] and t∈[1,T]. We also use boldface type to represent the sets or matrices of variables, e.g., variables of layer *l* at time step *t* are marked as $x_{l}^{t},u_{l}^{t},o_{l}^{t}$. Spike state space is marked as S. Notations E[·], P[·], p(·) and F(·) are, respectively, expectation, probability, probability distribution, and cumulative distribution function.

#### LIF neuron

We consider the commonly used LIF neuron model^91^^,^^92^ in this work, which describes the sub-threshold membrane potential dynamics as:

(Equation 3) $$\tau_{m}\frac{du}{dt}=-\left(u-u_{\text{reset}}\right)+RI\left(t\right),u<v_{\text{th}}\text{,}$$

where $R,\tau_{m}$ are membrane resistance and time constant, *I* is the input current, and $v_{\text{th}},u_{\text{reset}}$ are firing threshold and resting potential, respectively. It leads to the following discrete-time computational form in practice^7^^,^^93^:

(Equation 4) $$\begin{array}{l} \text{sub-threshold dynamic: }u^{t}=\tau u^{t-1}+\phi_{\theta }(x^{t}),\\ \text{firing: }o^{t}=\text{spike}(u^{t},v_{\text{th}})≜\text{Heaviside}(u^{t}-v_{\text{th}}),\\ \text{resetting: }u^{t}=u_{\text{reset}}\;if\;o^{t}=1, \end{array}$$

where $x^{t}$ is the input at time *t*, τ is the membrane time constant, and $\phi_{\theta }:S^{\dim\left(x^{t}\right)}\to R$ denotes a parameterized input transform. To introduce a simple model of neuronal spiking and refractoriness, we assume $v_{\text{th}}=1$, τ=0.5 and $u_{\text{reset}}=0$ throughout this research.

#### Noisy LIF neuron

The noisy LIF presented here is based on previous works that use diffusive approximation,^29^^,^^30^^,^^47^ where the sub-threshold dynamic is described by the Ornstein-Uhlenbeck process^94^^,^^95^:

(Equation 5) $$\begin{array}{l} \tau_{m}\frac{du}{dt}=-\left(u-u_{\text{reset}}\right)+RI\left(t\right)+\xi \left(t\right),e.q\text{.}\\ du=-\left(u-u_{\text{reset}}\right)\frac{dt}{\tau_{m}}+RI\left(t\right)\frac{dt}{\tau_{m}}+\sigma dW^{t}\text{,} \end{array}$$

where the white noise ξ is a stochastic process, σ is the amplitude of the noise, and $dW^{t}$ are the increments of the Wiener process in dt.^30^ As $\sigma dW^{t}$ are random variables drawn from a zero-mean Gaussian, this formulation is directly applicable to discrete-time simulations. Specifically, using the Euler-Maruyama method, we get a Gaussian noise term added on the right-hand side of Equation 4. Without loss of generality, we extend the additive noise term in the discrete form to general continuous noise,^96^ and by Equation 4, the sub-threshold dynamic of noisy LIF can be described as:

(Equation 6) $$\text{Noisy}\;\text{LIF}\;\text{sub}\text{-}\text{threshold}\;\text{dynamic}:u^{t}=\tau u^{t-1}+\phi_{\theta }\left(x^{t}\right)+\epsilon \text{,}$$

where the noise ϵ is independently drawn from a known distribution that satisfies E[ϵ]=0 and p(ϵ)=p(−ϵ). Equation 6 can also be obtained by discretizing an Itô stochastic differential equation variant of LIF neurons.^22^^,^^97^ We consider in this article Gaussian noise for all noisy LIF neurons in this study.

The membrane potentials and spike outputs become random variables due to the injection of random noises. Using noise as a medium, we naturally obtain the firing probability distribution of noisy LIF based on the threshold firing mechanism:

(Equation 7) $$\begin{array}{ccc} P[\text{firing at time }t] & = & P\underset{\text{Threshold firing}}{\underset{︸}{\left[u^{t}+\epsilon >v_{\text{th}}\right]}}\\ \; & = & \underset{\text{Cumulative Distribution Function definition}}{\underset{︸}{P[\epsilon <u^{t}-v_{\text{th}}]≜F_{\epsilon }(u^{t}-v_{\text{th}})}}\text{.} \end{array}$$

Therefore,

(Equation 8) $$o^{t}=\left\{\begin{array}{l} 1,\;\text{with probability}\;F_{\epsilon }(u^{t}-v_{\text{th}}),\;\\ 0,\;\text{with probability }\left(1-F_{\epsilon }(u^{t}-v_{\text{th}})\right), \end{array}\right)$$

The expressions above show how noise acts as a resource for computation.^46^ Thereby, we can formulate the firing process of noisy LIF as:

(Equation 9) $$\text{Noisy}\;\text{LIF}\;\text{probabilistic}\;\text{firing}:o^{t}∼\text{Bernoulli}\left(F_{\epsilon }\left(u^{t}-v_{\text{th}}\right)\right)\text{.}$$

Specifically, it relates to previous literature on escape noise models,^29^^,^^98^^,^^99^ in which the difference $u-v_{\text{th}}$ governs the neuron firing probabilities.^20^^,^^30^ In addition, noisy LIF employs the same resetting mechanism as the LIF model. Unless otherwise indicated, we focus on the discrete form in this research, which is of practical interest.

#### Noisy spiking neural network

**Figure 6:**
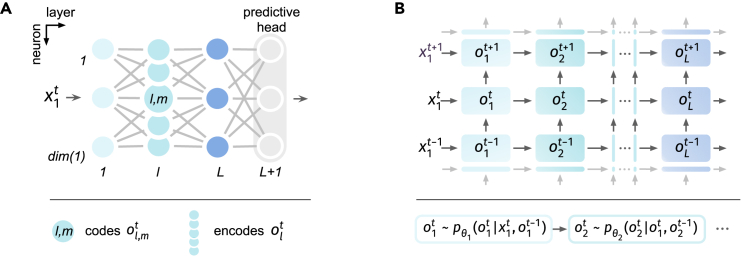
Graphical illustrations of NSNN (A) NSNN graphical illustration with neuron and layer notations, where $x_{1}$ denotes the initial input to the network. (B) Assumed dependencies between spike states using the Bayesian network frame. Taking time *t* as an example, the joint distribution is given by $p_{\theta }\left(o_{1\ldots L}^{t}|x_{1}^{t},o_{1\ldots L}^{t-1}\right)=p_{\theta_{1}}\left(o_{1}^{t}|x_{1}^{t},o_{1}^{t-1}\right)\prod_{l=2}^{L}p_{\theta_{l}}\left(o_{l}^{t}|o_{l-1}^{t},o_{l}^{t-1}\right)$, where θ denotes the synaptic parameters of the parametric model.

We consider the NSNN model as a probabilistic recognition model here as an example (Figure 6A). Let $x_{1}^{t}$ denote the input at time *t*, and using the dynamics of noisy LIF in Equations 6, 7, 8, and 9, an NSNN that consists of L+1 layers is given by:

(Equation 10) $$\text{input layer:}\;x_{1}^{t}=x_{1}^{t},\;u_{1}^{t}=\tau u_{1}^{t-1}+\Phi_{\theta_{1}}\left(x_{1}^{t}\right)+\epsilon_{1},\;o_{1}^{t}={\left\{o_{1,m}^{t}:o_{1,m}^{t}∼\text{Bernoulli}\left(P\left[o_{1,m}^{t}=1\right]\right)\right\}}_{m=1}^{dim\left(1\right)}\text{,}\;\text{hidden}\;\text{layer:}\;x_{l}^{t}=o_{l-1}^{t},\;u_{l}^{t}=\tau u_{l}^{t-1}+\Phi_{\theta_{l}}\left(x_{l}^{t}\right)+\epsilon_{l},\;o_{l}^{t}={\left\{o_{l,m}^{t}:o_{l,m}^{t}∼\text{Bernoulli}\left(P\left[o_{l,m}^{t}=1\right]\right)\right\}}_{m=1}^{dim\left(l\right)}\text{,}\;\text{predictive}\;\text{head:}\;L=f_{\theta_{L+1}}\left(o_{L}^{t}\right)=f\left(\Phi_{\theta_{L+1}}\left(o_{L}^{t}\right)\right)\text{.}\;\;\;\;\;\;\;$$

The output $o_{l}^{t}$ of layer *l* is a representation vector in the spike space $S^{\dim\left(l\right)}$, the membrane potentials $u_{l}^{t}\in R^{\dim\left(l\right)}$, and the mapping $\Phi_{\theta_{l}}:S^{\dim\left(l-1\right)}\to R^{\dim\left(l\right)}$. The noise vector $\epsilon_{l}\in R^{\dim\left(l\right)}$ consists of independent random noise with a known distribution (Gaussian in this article). The predictive head $f_{\theta_{L+1}}\left(o_{L}^{t}\right)$ includes a parameterized mapping $\varphi_{\theta_{L+1}}\left(o_{L}^{t}\right)$ and a loss function *f*, denoting the part that decodes predictions from the neural representation $o_{L}^{t}$ and computes the loss value. $\varphi_{\theta_{l}}$ represents a map like fully connected or convolution and is thus differentiable with respect to parameter $\theta_{l}$. Dividing the synaptic parameters by layers, as mentioned above, results in no loss of generality, as they can be defined as any differentiable mapping.

For example, to solve recognition problems, we shall consider the predictive probability model $p_{\theta_{L+1}}\left(y|o_{L}^{t}\right)=\text{softmax}\left(\phi_{\theta_{L+1}}\left(o_{L}^{t}\right)\right)$, where the map $\phi_{\theta_{L+1}}$ computes the predictive scores using the neural representation $o_{L}^{t}$. The function *f* can be the cross-entropy of the predictive distribution $p_{\theta_{L+1}}\left(y|o_{L}^{t}\right)$ and the target distribution $p_{\text{target}}\left(y|x_{1}^{t}\right)$. Note that $f_{\theta_{L+1}}\left(o_{L}^{t}\right)$ here computes the instantaneous loss, different from the $\frac{1}{T}\sum_{t}f^{t}$, which is computed over the entire time window and ignores potential online learning.^93^

Since each neuron codes for a random variable $o_{l,m}^{t}$, we can describe the NSNN by the Bayesian network model (Figure 6B) and represent the joint distribution of all spike states given input $x_{1}^{t}$ as:

(Equation 11) $$p_{\theta }\left(o_{1\ldots L}^{t}|x_{1}^{t},o_{1\ldots L}^{t-1}\right)=p_{\theta_{1}}\left(o_{1}^{t}|x_{1}^{t},o_{1}^{t-1}\right)\prod_{l=2}^{L}p_{\theta_{l}}\left(o_{l}^{t}|o_{l-1}^{t},o_{l}^{t-1}\right)\text{,}$$

where the layer representation is $p_{\theta_{l}}\left(o_{l}^{t}|o_{l-1}^{t},o_{l}^{t-1}\right)=\prod_{m=1}^{\dim\left(l\right)}p_{\theta_{l}}\left(o_{l,m}^{t}|o_{l-1}^{t},o_{l,m}^{t-1}\right)$.

#### Noise-driven learning

To perform NSNN synaptic optimization, the central problem is to estimate the gradient of the expected loss $E_{o_{1\cdots L}^{t}}\left[L\right]$. By Equations 10 and 11, $g_{l}$ is given by:

(Equation 12) $$g_{l}=\nabla_{\theta_{l}}E_{o_{1\cdots L}^{t}}\left[L\right]=\nabla_{\theta_{l}}\sum_{o_{1\ldots L}^{t}}p_{\theta }\left(o_{1\ldots L}^{t}|x_{1}^{t},o_{1\ldots L}^{t-1}\right)f_{\theta_{L+1}}\left(o_{L}^{t}\right)\text{.}$$

As Equation 12 is intractable to compute, we expect an estimation so that the parameters can be tuned using gradient-based routines.

The dimensionality of the spike-state space is rather limited (either spike or silence). Leveraging this property, we can derive an estimator by conditioning (local marginalization), which performs exact summation over a single random variable to reduce variance.^100^^,^^101^ We first factorize the joint distribution $p_{\theta }\left(o_{1\cdots L}^{t}|x_{1}^{t},o_{1\cdots L}^{t-1}\right)$ as the product of $\prod_{i\neq l}p_{\theta_{i}}\left(o_{i}^{t}|o_{i-1}^{t},o_{i}^{t-1}\right)$, $\prod_{k\neq m}p_{\theta_{l}}\left(o_{l,k}^{t}|o_{l-1}^{t},o_{l,k}^{t-1}\right)$, and $p_{\theta_{l}}\left(o_{l,m}^{t}|o_{l-1}^{t},o_{l,m}^{t-1}\right)$. Then, Equation 12 becomes:

(Equation 13) $$g_{l}=\sum_{o_{1\ldots L}^{t}}\sum_{m}\left(\prod_{i\neq l}p_{\theta_{i}}\left(o_{i}^{t}|o_{i-1}^{t},o_{i}^{t-1}\right)\prod_{k\neq m}p_{\theta_{l}}\left(o_{l,k}^{t}|o_{l-1}^{t},o_{l,k}^{t-1}\right)\right)\nabla_{\theta_{l}}p_{\theta_{l}}\left(o_{l,m}^{t}|o_{l-1}^{t},o_{l,m}^{t-1}\right)f_{\theta_{L+1}}\left(o_{L}^{t}\right)\text{.}$$

Using the fact $P\left[o_{l,m}^{t}=0\right]=1-P\left[o_{l,m}^{t}=1\right]$, we have:

(Equation 14) $$\sum_{o_{l,m}^{t}}\nabla_{\theta_{l}}p_{\theta_{l}}\left(o_{l,m}^{t}|o_{l-1}^{t},o_{l,m}^{t-1}\right)f_{\theta_{L+1}}\left(o_{L}^{t}\right)=\nabla_{\theta_{l}}p_{\theta_{l}}\left(o_{l,m}^{t}|o_{l-1}^{t},o_{l,m}^{t-1}\right)\Delta L\text{,}$$

where the loss difference term $\Delta L=f_{\theta_{L+1}}\left(o_{L}^{t}\right)-f_{\theta_{L+1}}\left(o_{\bar{l,m}}^{t}\right)$; here, we use $o_{\bar{l,m}}^{t}$ to denote the new state $o_{L}^{t}$ if $o_{l,m}^{t}$ changes. Given that $\sum_{o_{l,m}^{t}}p_{\theta_{l}}\left(o_{l,m}^{t}\right)=1$ and using Equations 13 and 14, we have:

(Equation 15) $$g_{l}=\sum_{o_{1\ldots L}^{t}}\left(\prod_{i=1}^{L}p_{\theta_{i}}\left(o_{i}^{t}|o_{i-1}^{t},o_{i}^{t-1}\right)\right){\hat{g}}_{l}=E_{o_{1\ldots L}^{t}}\left[{\hat{g}}_{l}\right]\text{,}$$

where

(Equation 16) $${\hat{g}}_{l}=\sum_{m}\nabla_{\theta_{l}}p_{\theta_{l}}\left(o_{l,m}^{t}|o_{l-1}^{t},o_{l,m}^{t-1}\right)\Delta L\text{.}$$

Intuitively, this local gradient is defined as a sum of the contributions of all neurons in layer *l*. To get an estimate of $g_{l}$, we can simply sample from $p_{\theta }\left(o_{1\cdots L}^{t}|x^{t}\right)$ and calculate using Equation 16. However, it is still unwise to compute ΔL as it requires repeated evaluations and, thus, cannot scale to large models.^55^ Inspired by previous studies,^55^^,^^102^^,^^103^ we may attribute the change of loss value to the state flip of variable $o_{l,m}^{t}$. By doing so, we may approximate the loss difference ΔL when the state of $o_{l,m}^{t}$ alters using a first-order approximation:

(Equation 17) $$\Delta L\approx \left(o_{l,m}^{t}-\left(1-o_{l,m}^{t}\right)\right)\frac{\partial f_{\theta_{L+1}}}{\partial o_{l,m}^{t}}=\left(2o_{l,m}^{t}-1\right)\nabla_{o_{l,m}^{t}}f_{\theta_{L+1}}\text{.}$$

This approximation introduces bias to the gradient estimator, except when the map *f* is multilinear.^55^^,^^103^ By Equations 16 and 17, we have:

(Equation 18) $${\hat{g}}_{l}=\sum_{m}\nabla_{\theta_{l}}p_{\theta_{l}}\left(o_{l,m}^{t}|o_{l-1}^{t},o_{l,m}^{t-1}\right)\left(2o_{l,m}^{t}-1\right)\nabla_{o_{l,m}^{t}}f_{\theta_{L+1}}\text{.}$$

By Equation 8, we have $\nabla_{\theta_{l}}p_{\theta_{l}}\left(o_{l,m}^{t}|o_{l-1}^{t},o_{l,m}^{t-1}\right)=\left(2o_{l,m}^{t}-1\right)F_{\epsilon }^{'}\left(u_{l,m}^{t}-v_{\text{th}}\right)\nabla_{\theta_{l}}u_{l,m}^{t}$. Therefore, by Equation 18, we can formulate the NDL rule as^52^^,^^53^:

(Equation 19) $$\text{NDL}:\;{\hat{g}}_{l}=\sum_{m}\underset{\text{Pre-synaptic}\;\text{factor}}{\underset{︸}{\nabla_{\theta_{l}}u_{l,m}^{t}}}\;\overset{\text{Post-synaptic}\;\text{factor}}{\overset{︷}{F_{\epsilon }^{'}\left(u_{l,m}^{t}-v_{\text{th}}\right)}}\underset{\text{Global}\;\text{learning}\;\text{signal}}{\underset{︸}{\nabla_{o_{l,m}^{t}}f_{\theta_{L+1}}}}\text{.}$$

Computing synaptic weights update using Equation 19 does not require calculating an additional gradient generator in the forward pass, and ${\hat{g}}_{l}$ can be computed layer by layer in a single backward passage. Specifically, the gradient estimation is performed by a backward pass, where the $\nabla_{u_{l,m}^{t}}o_{l,m}^{t}$ term in the exact chain rule is replaced by a term computed using the noise probability density function $F_{\epsilon }^{'}$. Therefore, NDL is easy to implement and can mesh well with modern automatic differentiation frameworks. And, since NDL is back-propagation compatible, we can use it to optimize NSNNs of any architecture easily.

Interestingly, some previous works^104^ also constructed a surrogate gradient function (pseudo-derivative to surrogate the $\nabla_{u_{l,m}^{t}}o_{l,m}^{t}$ term) by empirically adding infinitesimal Gaussian perturbations to the spiking neuron. The surrogate gradient function obtained by their single-neuron analysis shares similar insights compared with the post-synaptic term in NDL. However, our results are obtained from a network-level derivation.

### Theoretical analyses on internal noise and stability

**Figure 7:**
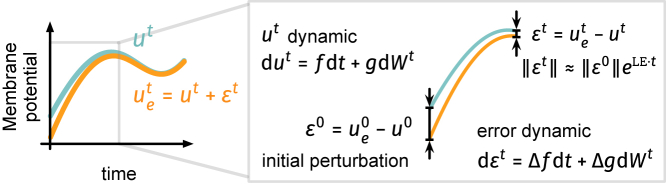
Illustration of the theoretical analysis We study the stability of the continuous NSNN sub-threshold dynamic (Equation 20) given a small initial perturbation $\epsilon^{0}$ by analyzing the stability of the error dynamic (Equation 22). If the trivial solution $\epsilon^{t}=0$ (with LE being the sample Lyapunov exponent) of the error dynamic is stable, the NSNN system can self-correct small perturbations, keeping the membrane potential in a controllable range and thus producing stable outputs.

Next, we analyze the stability of continuous NSNN sub-threshold dynamics (refer to Figure 7). Our analytical results show that the internal noise in NSNNs can benefit the stability against small perturbations by allowing faster self-correction. This will also offer another lens through which to highlight the potential of NSNNs for improved performance and robustness.^61^ To preface the analyses, we model an NSNN layer as a special case of a stochastic system consisting of one drift term f (to model the deterministic part) and one diffusion term g (to model the stochastic part), formally,

(Equation 20) $$du^{t}=f\left(u^{t},I^{t}\right)dt+g\left(u^{t},I^{t}\right)dW^{t}\text{,}$$

where $u^{t},I^{t}\in R^{\dim\left(u^{t}\right)}$, $W^{t}$ is a $\dim\left(u^{t}\right)$-dimensional Wiener process, and the coefficients f,g satisfy the following assumption.

#### Assumption

There exists a constant K>0 such that $∥f\left(u_{1}^{t},t\right)-f\left(u_{2}^{t},t\right)∥+∥g\left(u_{1}^{t},t\right)-g\left(u_{2}^{t},t\right)∥\leq K∥u_{1}^{t}-u_{2}^{t}∥$ for all $u_{1}^{t},u_{2}^{t}\in R^{\dim\left(u^{t}\right)},t\in R^{+}$.

In particular, we focus on the choice of f and g:

(Equation 21) $$\begin{array}{l} f\left(u^{t},I^{t}\right)=a_{1}u^{t}+B_{1}I^{t}\text{,}\\ g\left(u^{t},I^{t}\right)=a_{2}\text{diag}\left(1\right)+b_{2}f\left(u^{t},I^{t}\right)\text{,} \end{array}$$

with constants $a_{1}\in R$, $a_{2},b_{2}\in [0,\infty )$ and matrix $B_{1}\in R^{\dim\left(u^{t}\right)\times \dim\left(u^{t}\right)}$. We can recover an NSNN layer consisting of noisy LIF neurons described in the main text by letting $a_{1}=-1/\tau_{m}$, $a_{2}=\sigma$, and $b_{2}=0$.

Further, consider initializing the system represented by Equations 20 and 21 with two slightly different initializations $u^{0}$, $u_{e}^{0}≜u^{0}+\epsilon^{0}$, where $\epsilon^{0}\in R^{\dim\left(u\right)}$ is the initial external perturbation (error) on membrane potentials. Then, the evolution of error $\epsilon^{t}=u_{e}^{t}-u^{t}$ satisfies the stochastic differential equation (SDE):

(Equation 22) $$d\epsilon^{t}=\Delta f\left(\epsilon^{t}\right)dt+\Delta g\left(\epsilon^{t}\right)dt\text{,}$$

where

(Equation 23) $$\begin{array}{l} \Delta f\left(\epsilon^{t}\right)≜\left(f\left(u^{t}+\epsilon^{t},I^{t}\right)-f\left(u^{t},I^{t}\right)\right)\text{,}\\ \Delta g\left(\epsilon^{t}\right)≜\left(g\left(u^{t}+\epsilon^{t},I^{t}\right)-g\left(u^{t},I^{t}\right)\right)\text{.} \end{array}$$

Here we assume that the two random processes $u^{t},u_{e}^{t}=u^{t}+\epsilon^{t}$ are driven by the same Wiener process to make the subtraction operation valid. Since Δf(0)=0 and Δg(0)=0, $\epsilon^{t}=0$ admits a trivial solution for Equation 22, whose uniqueness is guaranteed by the above assumption.^105^ We focus on analyzing the stability of the trivial solution $\epsilon^{t}=0$; if it is stable, the small initial perturbation $\epsilon^{0}\neq 0$ will be reduced as the system evolves. This means that the system described by Equation 20 is self-correcting and can function reliably in the face of small perturbations or errors. Specifically, we focus on the “almost sure exponential stability” defined as follows.

#### Almost sure exponential stability^105^

The trivial solution $\epsilon^{t}=0$ is almost surely exponentially stable if the sample Lyapunov exponent $LE≜{\lim\;\sup}_{t\to \infty }\frac{1}{t}\log∥\epsilon^{t}∥$ is almost surely negative for all $\epsilon^{0}\in R^{\dim\left(u^{t}\right)}$.

With LE being the sample Lyapunov exponent of the trivial solution, there exists a positive constant *C* and a random variable τ∈[0,∞) such that for all t>τ, $∥\epsilon^{t}∥\leq C\;\exp\;\left(t\cdot LE\right)$ with probability 1. Therefore, the almost sure exponential stability implies that almost all sample paths starting from non-zero initializations will tend to the equilibrium position $\epsilon^{t}=0$ exponentially fast, i.e., the NSNN system can quickly self-correct small perturbations. Further, we have an important result regarding the bounds of the sample Lyapunov exponent.

#### Theorem 1: Bounds for sample Lyapunov exponent of the trivial solution $\epsilon^{t}=0$

Suppose that $0\leq a_{2}∥\epsilon^{t}∥\leq ∥\Delta g\left(\epsilon^{t}\right){∥}_{F}\leq \left(a_{2}+b_{2}\right)∥\epsilon^{t}∥$ for all non-zero $\epsilon^{t}\in R^{\dim\left(u^{t}\right)}$, $t\in R^{+}$. Then, almost surely,

$$a_{1}-\frac{1}{2}a_{2}^{2}-b_{2}^{2}-2a_{2}b_{2}\leq LE\leq a_{1}-\frac{1}{2}a_{2}^{2}+\frac{1}{2}b_{2}^{2}+a_{2}b_{2}$$

for all $\epsilon^{0}\in R^{\dim\left(u^{t}\right)}$.

To prove Theorem 1, we first introduce the differential operator *L*. And for the brevity of notations, we temporally omit the superscript *t*, viz., we use u,ϵ rather than $u^{t},\epsilon^{t}$ here. The differential operator associated with Equation 22 is defined by:

(Equation 24) $$L=\frac{\partial }{\partial t}+\sum_{i=1}^{\dim\left(u\right)}\Delta f_{i}\left(\epsilon ,t\right)\frac{\partial }{\partial \epsilon_{i}}+\frac{1}{2}\sum_{i,j=1}^{\dim\left(u\right)}{\left[\Delta g^{⊤}\left(\epsilon ,t\right)\Delta g\left(\epsilon ,t\right)\right]}_{ij}\frac{\partial^{2}}{\partial \epsilon_{i}\partial \epsilon_{j}}\text{,}$$

where the subscripts denote the entries of tensors, and we also introduce a lemma as follows.

#### Lemma: Stochastic Lyapunov theorem

Assume that there exists a non-negative real valued function V(ϵ,t) defined on $R^{\dim\left(u\right)}\times R^{+}$, denoted as $V\in C^{2,1}\left(\dim\left(u\right)\times R^{+};R^{+}\right)$. The function *V* has continuous partial derivatives denoted as:

$$V_{\epsilon }=\frac{\partial V}{\partial \epsilon },V_{t}=\frac{\partial V}{\partial t},V_{\epsilon ,\epsilon }=\frac{\partial^{2}V}{\partial \epsilon \partial \epsilon^{⊤}}\text{,}$$

and constants $c_{1},C_{1}>0$, $c_{2},C_{2}\in R$, $c_{3},C_{3}\geq 0$ such that for all non-zero ϵ and $t\in R^{+}$:

$$\left(1\right)\;c_{1}∥\epsilon {∥}^{2}\leq V\left(\epsilon ,t\right)\leq C_{1}∥\epsilon {∥}^{2},$$

$$\left(2\right)\;c_{2}V\left(\epsilon ,t\right)\leq LV\left(\epsilon ,t\right)\leq C_{2}V\left(\epsilon ,t\right),$$

$$\left(3\right)\;2c_{3}V{\left(\epsilon ,t\right)}^{2}\leq ∥V_{\epsilon }\left(\epsilon ,t\right)\Delta g\left(\epsilon \right){∥}_{F}^{2}\leq C_{3}V\left(\epsilon ,t\right),$$

Then, the sample Lyapunov exponent satisfies

$$\frac{c_{2}}{2}-\frac{C_{3}}{4}\leq \underset{t\to \infty }{\lim\;\sup}\frac{1}{t}\log∥\epsilon ∥\leq \frac{C_{2}}{2}-\frac{c_{3}}{4}\;a.s\text{.}$$

This Lemma can be proved by combining Theorems 3.3 and 3.5 in Mao,^105^ Chapter 4, with p=2 as in Lim et al.^25^ With this Lemma in tow, we then prove Theorem 1.

#### Proof of Theorem 1

Let $V\left(\epsilon ,t\right)=∥\epsilon {∥}^{2}$. If we assign $c_{1}=C_{1}=1$, condition 1 in the Lemma is satisfied. By Equation 24, we have:

$$LV\left(\epsilon ,t\right)=V_{\epsilon }^{⊤}\Delta f\left(\epsilon ,t\right)+\frac{1}{2}\text{trace}\left(\Delta g^{⊤}\left(\epsilon ,t\right)V_{\epsilon ,\epsilon }\Delta g\left(\epsilon ,t\right)\right)\text{.}$$

Then, by Equations 21 and 22, we can easily show that, for all non-zero $\epsilon \in R^{\dim\left(u\right)}$, $t\in R^{+}$:

$$\left(2a_{1}+a_{2}^{2}\right)∥\epsilon {∥}^{2}\leq LV\left(\epsilon ,t\right)\leq \left(2a_{1}+{\left(a_{2}+b_{2}\right)}^{2}\right)∥\epsilon {∥}^{2}\text{.}$$

Hence, condition 2 in the Lemma is satisfied.

To satisfy the condition 3 in the Lemma, by the assumption on Δg, we have:

$$4a_{2}^{2}V^{2}\leq ∥V_{\epsilon }\Delta g{∥}_{F}^{2}\leq 4{\left(a_{2}+b_{2}\right)}^{2}V^{2}\text{.}$$

In conclusion, let $c_{1}=1,C_{1}=1$, $c_{2}=2a_{1}+a_{2}^{2},C_{2}=2a_{1}+{\left(a_{2}+b_{2}\right)}^{2}$, $c_{3}=4a_{2}^{2},C_{3}=4{\left(a_{2}+b_{2}\right)}^{2}$, and we obtain the bounds of the sample Lyapunov exponent by the Lemma, that is:

$$a_{1}-\frac{1}{2}a_{2}^{2}-b_{2}^{2}-2a_{2}b_{2}\leq LE\leq a_{1}-\frac{1}{2}a_{2}^{2}+\frac{1}{2}b_{2}^{2}+a_{2}b_{2},\text{ Q.E.D.}$$

According to Theorem 1, the lower bound, LB, and upper bound, UB, of the sample Lyapunov exponent of the trivial solution $\epsilon^{t}=0$ are:

$$LB=a_{1}-\frac{1}{2}a_{2}^{2}-b_{2}^{2}-2a_{2}b_{2},UB=a_{1}-\frac{1}{2}a_{2}^{2}+\frac{1}{2}b_{2}^{2}+a_{2}b_{2}\text{.}$$

Generally, by the definition of almost sure exponential stability, if UB<0, the trivial solution is almost surely exponentially stable. And even if $a_{1}>0$, the system can still be stabilized by introducing internal noise terms to ensure a negative upper bound. For LIF-modeling SNNs, as $a_{1}=-\frac{1}{\tau_{m}}<0$, if $\frac{1}{\tau_{m}}>-\frac{1}{2}a_{2}^{2}+\frac{1}{2}b_{2}^{2}+a_{2}b_{2}$, then we have that the sub-threshold dynamic system is self-correcting, i.e., $\epsilon^{t}\overset{a.s.}{\to }0$. We then turn to the special case when the noise is additive, $\left(a_{2}>0,b_{2}\to 0\right)$. NSNNs introduced in this article belong to this category. In the additive noise case, the multiplicative noise component in the system tends to zero, $b_{2}\to 0$, and the upper bound of the sample Lyapunov exponent becomes $UB_{\text{additive}}=a_{1}-\frac{1}{2}a_{2}^{2}$. On the other hand, in the noiseless case, both noise terms tend to zero, $a_{2}\to 0,b_{2}\to 0$, and the lower bound is $LB_{\text{noise}\;\text{free}}=a_{1}$. Therefore we have $UB_{\text{additive}}<LB_{\text{noise}\;\text{free}}=a_{1}<0$. In a small time interval, for the error at time *t*, we have $∥\epsilon^{t}∥=∥\epsilon^{0}∥\exp\;\left(LE\cdot t\right)$. Therefore, introducing additive noise ensures a more negative Lyapunov exponent than in the noiseless case, resulting in faster self-correction. This allows rapid detection and correction of factors that may cause instability, thereby improving NSNN system stability.

### Experimental details

Experiments were conducted using a workstation with an Intel I5-10400, 64 GB RAM, and two NVIDIA RTX 3090s. The results are reported as mean and standard deviation (SD) across multiple independent runs.

#### Details of recognition experiments

The CIFAR dataset^58^ includes 50,000 32×32 images for training and 10,000 for evaluation. We adopt random crop, random horizontal flip, and AutoAugment^106^ for the training samples. The pre-processed samples are normalized using *Z*-score scaling in the training and evaluation phases. The DVS-CIFAR dataset^59^ is a challenging neuromorphic benchmark recorded via a DVS camera using images from CIFAR-10. We adopt a pre-processing pipeline following previous works,^107^ i.e., divide the original set into a 9,000-sample training set and 1,000-sample evaluation set, and all event stream files are spatially downsampled to 48×48. We augment the training samples following previous studies.^9^ The DVS-Gesture dataset^60^ is recorded using a DVS128 event camera. It contains recordings of 11 hand gestures from 29 subjects under three illumination conditions.

We optimized SNN models using the dataset’s observation-label pairs and calculated performance metrics on non-overlapping test data. Prediction accuracy was used as the performance metric for these tasks. For each network architecture and SNN algorithm combination, we set the optimal hyperparameters for DSNNs and NSNNs under different architecture and algorithm combinations via grid search to ensure a fair comparison. We indicate the corresponding simulation time step in the results, which is the simulation duration of our discrete SNN implementation. The static image is repeatedly inputted for static-image data, so a longer simulation time step usually results in more accurate recognition. For event-stream datasets, the fixed-length continuous event streams were discretized into simulation time-step time windows. The information embedded in those event streams will be spread over many time steps. Hence, a larger simulation time step usually leads to more refined computation and more accurate recognition.

We set the SD of membrane noise to 0.3 for CIFAR-10, CIFAR-100, and DVS-Gesture experiments and 0.2 for DVS-CIFAR ones. These configurations offer a fair balance between performance and resilience. For the SGL of DSNNs, we employ the ERF surrogate gradient ${\text{SG}}_{\text{ERF}}\left(x\right)=\frac{1}{\sqrt{\pi }}\;\exp\;\left(-x^{2}\right)$. Adam solvers^108^ with the cosine annealing learning rate scheduler^109^ was used to train all networks. We list hyperparameters we adopted in recognition experiments in Table 4. The initial learning rate is obtained through a grid search. The ResNet-19,^110^ VGGSNN,^9^ CIFARNet,^8^ and 7B-Net^12^ SNN architectures used in this article follow the original implementation in previous works. The ResNet-18 architecture is given by 64C3-2(64C3-4C3)-2(128C3-128C3)-2(256C3-256C3)-2(512C3-512C3)-AP-FC. AP denotes average pooling, FC denotes the fully connected layer, and C denotes the convolution layer. The SNN algorithms STCA,^111^ STBP,^7^ STBP-tdBN^110^ (tdBN), and TET^9^ used here follow the original implementations presented in these works. Some performances reported in the comparison come from the original paper, including those of LIAF-Net,^112^ STCA,^111^ STBP-tdBN^110^ with ResNet-19 and ResNet-17, TET^9^ with ResNet-19, Wide-7B-Net,^12^ and 7B-Net.^12^

**Table 4:** Hyperparameter settings for recognition experiments

NSNN	Data	SNN algorithm	SNN architecture	Simulation time step	Initial learning rate	Mini-batch size
–	CIFAR-10^58^	STBP^7^	ResNet-18	2/4	0.01	256/256
–	CIFAR-10	TET^9^	ResNet-18	2/4	0.01	256/256
–	CIFAR-10	STBP	CIFARNet	2/4	0.004	256/256
–	CIFAR-100^58^	STBP	ResNet-18	2/4	0.005	256/256
–	CIFAR-100	TET	ResNet-18	2/4	0.005	256/256
–	CIFAR-100	STBP	CIFARNet	2/4	0.001	256/256
–	DVS-CIFAR^59^	STBP	ResNet-19	10	0.0005	32
–	DVS-CIFAR	TET	VGGSNN	10	0.0002	64
–	DVS-CIFAR	tdBN^110^	VGGSNN	10	0.0002	64
–	DVS-Gesture^60^	STBP	7B-Net	16	0.001	10
Yes	CIFAR-10	STBP	ResNet-18	2/4	0.002	256/256
Yes	CIFAR-10	TET	ResNet-18	2/4	0.002	256/256
Yes	CIFAR-10	STBP	CIFARNet	2/4	0.003	256/128
Yes	CIFAR-100	STBP	ResNet-18	2/4	0.001	256/256
Yes	CIFAR-100	TET	ResNet-18	2/4	0.001	256/256
Yes	CIFAR-100	STBP	CIFARNet	2/4	0.002	256/128
Yes	DVS-CIFAR	STBP	ResNet-19	10	0.0005	20
Yes	DVS-CIFAR	TET	VGGSNN	10	0.0003	32
Yes	DVS-CIFAR	tdBN	VGGSNN	10	0.0003	32
Yes	DVS-Gesture	STBP	7B-Net	16	0.0005	8

**Figure 8:**
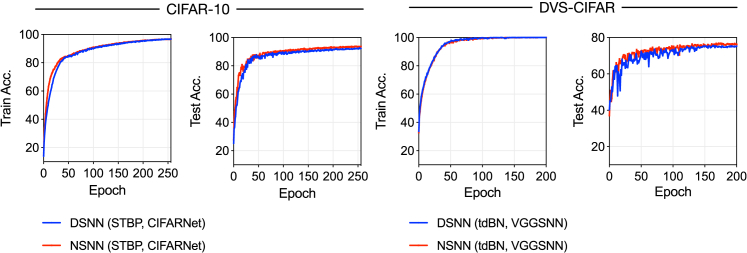
Example learning curves on CIFAR-10 and DVS-CIFAR

We visualize representative learning curves of DSNNs and NSNNs. As seen from Figure 8, NSNNs demonstrated higher learning efficiency than DSNNs. And this advantage is more pronounced on datasets prone to overfitting, such as the DVS-CIFAR data. We also noticed that using NSNNs results in a slight increase in training time compared with DSNNs. This is mainly because the firing process in noisy LIF neurons involves computing the firing probability and sampling from the Bernoulli distribution. Much of this additional time comes from the sampling step, since the PyTorch framework we use has no targeted optimizations for sampling from random distributions. For instance, training a CIFARNet on CIFAR-10 data takes about 183 min for DSNNs and 194 min for NSNNs.

#### Effect of internal noise level on network performance

Although the noise-based computation and learning approaches were presented before, one question remains: how do we choose an appropriate noise scale for the NSNNs? The internal noise level influences learning, as the post-synaptic factor ${F^{'}}_{\epsilon }\left(u-v_{\text{th}}\right)$ in NDL is calculated using the probability density function of membrane noise ϵ. When the variance is tiny, the noise distribution converges to a Dirac distribution with limited information (as measured by information entropy), preventing the post-synaptic factor from obtaining enough information to perform learning effectively. In the case of inference, the noise level directly influences the randomness of the firing distribution; smaller noise causes less corruption of the neuronal dynamics, while larger noise (with high variance) would overcorrupt the membrane voltage dynamics, thereby disrupting the flow of decisive information in the network, leading to greatly deteriorated performance.

The previous content provided a theoretical explanation regarding internal noise, network stability, and performance. Here we show empirically that mild noise is beneficial for network performance. In early theoretical research,^61^ through the analysis of neuronal dynamics, researchers pointed out that, under certain conditions, noise can have a positive impact on performance. More recent literature^113^ studied the learning performance of small-scale spiking neural models with neuronal white noise under the STDP learning rule. It was found that appropriate levels of neuronal noise could benefit the model’s learning ability, which is consistent with the experimental results we obtained from our quantitative analysis in this section.

**Figure 9:**
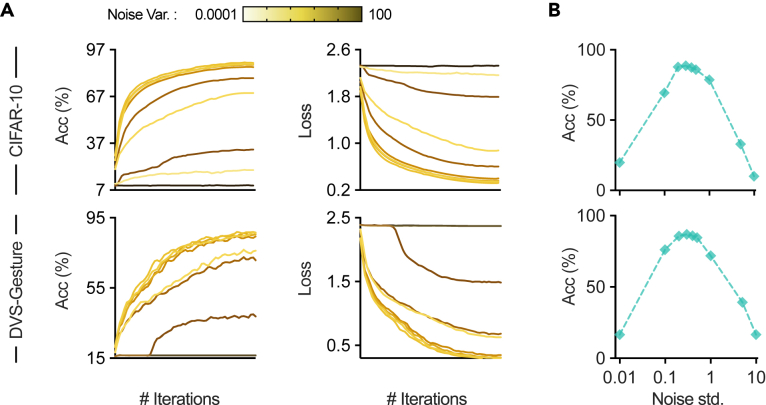
Effects of internal noise level on performance (A) Learning curves of NSNNs under different noise levels. We use color to distinguish different noise levels. (B) The relationship between final test accuracy and the standard deviation of internal noise ϵ.

We ran experiments using the CIFAR-10 and DVS-Gesture datasets and trained identical networks with different internal noise level settings for 60 epochs. Results are presented by learning curves and the accuracy-SD curves in Figure 9A. Our observations show that, when the variance of internal noise increases from 0, the model’s performance initially improves and then declines. Notably, NSNNs achieve high performance near a moderate value (refer to Figure 9B), confirming our intuition that moderate noise is essential for high performance. According to our results, changes in std[ϵ] within a *moderate noise* range (from 0.2 to 0.5) have no significant effect on final performance. This gives us a range of internal noise levels to choose from when using NSNNs in practice.

#### Details of recognition experiments with perturbations

Here we list the details of the external perturbations in our experiments. We denote the model to be evaluated as NN. In the DO method, we construct the adversarial samples by directly solving the constrained optimization problem:

(Equation 25) $$\Delta x={\mathrm{argmax}}_{{\left\|\Delta x\right\|}_{2}=\gamma }\text{loss}\left(f\left(x+\Delta x\right),y\right)\text{,}$$

where Δx is for the adversarial perturbation and x+Δx is the adversarial example. It is implemented using PyTorch and GeoTorch^114^ toolkits. The L-2 norm-bounded additive disturbance tensors are zero-initialized and optimized by an Adam solver with a learning rate of 0.002 for 30 iterations. After that, the additive perturbations are used to produce adversarial samples and fed into the target models (DSNNs or NSNNs in this work). The implementation of the FGSM follows the original implementation,^115^ where the adversarial example is constructed as:

(Equation 26) $${\tilde{x}}^{adv}=x+\gamma_{FGSM}\times \text{sign}\left[\nabla_{x}\text{loss}\left(\text{NN}\left(x\right),y\right))\right]\text{.}$$

The input-level EventDrop^63^ perturbation for dynamic inputs is constructed by randomly dropping spikes in the raw input spike trains. The dropping probability is set by a parameter ρ. The strategy of dropping we consider is Random Drop,^63^ which combines spatial and temporal-wise event-dropping strategies. During the evaluation, we first individually performed EventDrop over every sample from the test set and then fed our testees with the disturbed inputs.

The spike-state-level perturbation includes two disturbances: the emission state from 1 to 0 (spike to silence) and the emission state from 0 to 1 (silence to spike). To simplify the settings, we use one parameter β, to control the probability of both kinds of disturbances. Let variable $o_{\text{old}}$ denote the original spike state; if $o_{\text{old}}=1$, we have $P\left[o_{\text{new}}=0\right]=\beta$, else, if $o_{\text{old}}=0$, $P\left[o_{\text{new}}=1\right]=\beta$.

#### Details of recognition task coding analyses

In this part, we used the learned models in previous recognition experiments, whose simulation time steps are 2, 2, 10, and 16, respectively. The number of test samples for computing the Pearson correlation coefficients is 500 for CIFAR-10, CIFAR-100, and DVS-CIFAR and 200 for DVS-Gesture. In our analyses, the spike count variability is measured by the FF,^67^^,^^116^ which is calculated as follows: let $n_{L,m}^{\text{trial}\;\text{ID}}$ be the spike count of neuron (L,m), and the mean value is ${\bar{n}}_{L,m}=\frac{1}{\#\;\text{trials}}\sum_{k}n_{L,m}^{k}$; the deviations from that mean is computed as $\Delta n_{L,m}^{\text{trial}\;\text{ID}}=n_{L,m}^{\text{trial}\;\text{ID}}-{\bar{n}}_{L,m}$, then, FF is given by ${\text{FF}}_{L,m}=\frac{\text{Var}\left[n_{L,m}\right]}{{\bar{n}}_{L,m}}$.

#### Details of neural activity fitting experiments

In the neural activity fitting experiments, we used neural recordings of dark-adapted axolotl salamander retinas.^68^ The original dataset contains the retinal neural responses of two retinas to two movies. We used only a part of the data (responses of retina 2 to movie 2) in this experiment. It contained complex natural scenes of a tiger on a prey hunt and was roughly 60 s long. The movie was discretized into bins of 33 ms, and all frames were converted to gray scale with a resolution of 360 × 360 pixels at 7.5 × 7.5 μm per pixel, covering a 2,700 × 2,700 μm area on the retina. The neural recording contained 42 repetitions of 49 cells. We partitioned all records into stimulus-response sample pairs of 1 s and downsampled the frames to 90 × 90 pixels. The data were split into non-overlapping train/test (50%/50%) parts.

The network architecture for the DSNN and NSNN models is 16C25-32C11-FC64-FC64-FC64-FC49. FC denotes the fully connected layer, and C denotes the convolutional layer. These models were trained using the Adam optimizer with a cosine-decay learning rate scheduler, starting at a rate of 0.0003. The mini-batch size was set to 64, and the models were trained for 64 epochs. At test time, the test samples had the same length as the training samples (1 s) by default. During training, the DSNN and NSNN models were optimized to minimize the maximum mean discrepancy (MMD) loss.^117^^,^^118^ We use the first-order post-synaptic potential (PSP) kernel,^119^ which can effectively depict the temporal dependencies in spike train data. Denoting the predicted and recorded spike trains as $\hat{y}$ and y, respectively, we can write the PSP kernel MMD predictive loss as:

(Equation 27) $$L_{\text{PSP}-\text{MMD}}=\frac{1}{T}\sum_{t=1}^{T}\sum_{\tau =1}^{t}∥\text{PSP}\left({\hat{y}}_{1:\tau }\right)-\text{PSP}\left(y_{1:\tau }\right){∥}^{2}\text{,}$$

where $\text{PSP}\left(y_{1:\tau }\right)=\left(1-\frac{1}{\tau_{s}}\right)\text{PSP}\left(y_{1:\tau -1}\right)+\frac{1}{\tau_{s}}y_{\tau }$, and we set the time constant as $\tau_{s}=2$. For the DSNN model, we used the ERF surrogate gradient as in the recognition experiments. And for the NSNN model, the internal noise was $N\left(0,0.2^{2}\right)$.

The CNN baseline we used adopts the architecture 32C25-BN-16C11-BN-FC49-BN-PSoftPlus, where BN denotes the batch-normalization operation, and PSoftPlus denotes the parameterized SoftPlus activation. The training specifications follow the implementation in the previous work.^69^

## Data Availability

The databases used in this paper are publicly available and can be accessed as follows. CIFAR-10, and CIFAR-100: https://www.cs.toronto.edu/∼kriz/cifar.html, DVS-Gesture (DVS128 Gesture): https://research.ibm.com/interactive/dvsgesture/, DVS-CIFAR (CIFAR10-DVS): https://figshare.com/s/d03a91081824536f12a8. The codebase^90^ can be found at github.com/genema/Noisy-Spiking-Neuron-Nets or gitee.com/ghma/Noisy-Spiking-Neuron-Nets. Any additional information required to reanalyze the data reported in this paper is available from the lead contact upon request.

## References

[1] Zilli E.A., Hasselmo M.E. (2010). Coupled noisy spiking neurons as velocity-controlled oscillators in a model of grid cell spatial firing. J. Neurosci..

[2] Teeter C., Iyer R., Menon V., Gouwens N., Feng D., Berg J., Szafer A., Cain N., Zeng H., Hawrylycz M. (2018). Generalized leaky integrate-and-fire models classify multiple neuron types. Nat. Commun..

[3] Simonyan K., Zisserman A. (2014). Very deep convolutional networks for large-scale image recognition. arXiv.

[4] Szegedy C., Liu W., Jia Y., Sermanet P., Reed S., Anguelov D., Erhan D., Vanhoucke V., Rabinovich A. (2015). The IEEE/CVF Computer Vision and Pattern Recognition Conference.

[5] He K., Zhang X., Ren S., Sun J. (2016). The IEEE/CVF Computer Vision and Pattern Recognition Conference.

[6] Lee J.H., Delbruck T., Pfeiffer M. (2016). Training deep spiking neural networks using backpropagation. Front. Neurosci..

[7] Wu Y., Deng L., Li G., Zhu J., Shi L. (2018). Spatio-temporal backpropagation for training high-performance spiking neural networks. Front. Neurosci..

[8] Wu Y., Deng L., Li G., Zhu J., Xie Y., Shi L. (2019). Proceedings of the AAAI conference on artificial intelligence.

[9] Deng S., Li Y., Zhang S., Gu S. (2021). International Conference on Learning Representations.

[10] Volinski A., Zaidel Y., Shalumov A., DeWolf T., Supic L., Ezra Tsur E. (2022). Data-driven artificial and spiking neural networks for inverse kinematics in neurorobotics. Patterns.

[11] Zhao F., Zeng Y., Han B., Fang H., Zhao Z. (2022). Nature-inspired self-organizing collision avoidance for drone swarm based on reward-modulated spiking neural network. Patterns.

[12] Fang W., Yu Z., Chen Y., Huang T., Masquelier T., Tian Y. (2021). Advances in Neural Information Processing Systems.

[13] Liu Q., Ruan H., Xing D., Tang H., Pan G. (2020). Proceedings of the AAAI conference on artificial intelligence.

[14] Roy K., Jaiswal A., Panda P. (2019). Towards spike-based machine intelligence with neuromorphic computing. Nature.

[15] Verveen A.A., DeFelice L.J. (1974). Membrane noise. Prog. Biophys. Mol. Biol..

[16] Kempter R., Gerstner W., Van Hemmen J.L., Wagner H. (1998). Extracting oscillations: Neuronal coincidence detection with noisy periodic spike input. Neural Comput..

[17] Stein R.B. (1965). A theoretical analysis of neuronal variability. Biophys. J..

[18] Stein R.B., Gossen E.R., Jones K.E. (2005). Neuronal variability: noise or part of the signal?. Nat. Rev. Neurosci..

[19] Faisal A.A., Selen L.P.J., Wolpert D.M. (2008). Noise in the nervous system. Nat. Rev. Neurosci..

[20] Maass W. (1995). Advances in Neural Information Processing Systems.

[21] Maass W. (1996). Advances in Neural Information Processing Systems.

[22] Patel A., Kosko B. (2005). Stochastic resonance in noisy spiking retinal and sensory neuron models. Neural Network..

[23] Liu X., Xiao T., Si S., Cao Q., Kumar S., Hsieh C.-J. (2020). The IEEE/CVF Computer Vision and Pattern Recognition Conference.

[24] Camuto A., Willetts M., Simsekli U., Roberts S.J., Holmes C.C. (2020).

[25] Lim S.H., Erichson N.B., Hodgkinson L., Mahoney M.W. (2021). Advances in Neural Information Processing Systems.

[26] Hinton G.E., Srivastava N., Krizhevsky A., Sutskever I., Salakhutdinov R.R. (2012). Improving neural networks by preventing co-adaptation of feature detectors. arXiv.

[27] Gerstein G.L., Mandelbrot B. (1964). Random walk models for the spike activity of a single neuron. Biophys. J..

[28] Tuckwell H.C. (1989).

[29] Plesser H.E., Gerstner W. (2000). Noise in integrate-and-fire neurons: from stochastic input to escape rates. Neural Comput..

[30] Gerstner W., Kistler W.M., Naud R., Paninski L. (2014).

[31] Rao R.P.N. (2004). Bayesian computation in recurrent neural circuits. Neural Comput..

[32] Rao R.P. (2004).

[33] Deneve S. (2004).

[34] Kasabov N. (2010). To spike or not to spike: A probabilistic spiking neuron model. Neural Network..

[35] Skatchkovsky N., Jang H., Simeone O. (2021). Spiking neural networks—part ii: Detecting spatio-temporal patterns. IEEE Commun. Lett..

[36] Neftci E.O., Mostafa H., Zenke F. (2019). Surrogate gradient learning in spiking neural networks: Bringing the power of gradient-based optimization to spiking neural networks. IEEE Signal Process. Mag..

[37] Cramer B., Billaudelle S., Kanya S., Leibfried A., Grübl A., Karasenko V., Pehle C., Schreiber K., Stradmann Y., Weis J. (2022). Surrogate gradients for analog neuromorphic computing. Proc. Natl. Acad. Sci. USA.

[38] Eshraghian J.K., Ward M., Neftci E., Wang X., Lenz G., Dwivedi G., Bennamoun M., Jeong D.S., Lu W.D. (2021). Training spiking neural networks using lessons from deep learning. arXiv.

[39] Bellec G., Scherr F., Subramoney A., Hajek E., Salaj D., Legenstein R., Maass W. (2020). A solution to the learning dilemma for recurrent networks of spiking neurons. Nat. Commun..

[40] Rumelhart D.E., Hinton G.E., Williams R.J. (1986). Learning representations by back-propagating errors. Nature.

[41] Zenke F., Vogels T.P. (2021). The remarkable robustness of surrogate gradient learning for instilling complex function in spiking neural networks. Neural Comput..

[42] Jang H., Simeone O., Gardner B., Gruning A. (2019). An introduction to probabilistic spiking neural networks: Probabilistic models, learning rules, and applications. IEEE Signal Process. Mag..

[43] Dan Y., Poo M.-m. (2004). Spike timing-dependent plasticity of neural circuits. Neuron.

[44] Froemke R.C., Poo M.-m., Dan Y. (2005). Spike-timing-dependent synaptic plasticity depends on dendritic location. Nature.

[45] Guyonneau R., VanRullen R., Thorpe S.J. (2005). Neurons tune to the earliest spikes through stdp. Neural Comput..

[46] Maass W. (2014). Noise as a resource for computation and learning in networks of spiking neurons. Proc. IEEE.

[47] Burkitt A.N. (2006). A review of the integrate-and-fire neuron model: I. homogeneous synaptic input. Biol. Cybern..

[48] Heckerman D., Geiger D., Chickering D.M. (1995). Learning bayesian networks: The combination of knowledge and statistical data. Mach. Learn..

[49] Heckerman D. (1998).

[50] Zenke F., Neftci E.O. (2021). Brain-inspired learning on neuromorphic substrates. Proc. IEEE.

[51] Wu Y., Zhao R., Zhu J., Chen F., Xu M., Li G., Song S., Deng L., Wang G., Zheng H. (2022). Brain-inspired global-local learning incorporated with neuromorphic computing. Nat. Commun..

[52] Frémaux N., Gerstner W. (2016). Neuromodulated spike-timing-dependent plasticity, and theory of three-factor learning rules. Front. Neural Circ..

[53] Gerstner W., Lehmann M., Liakoni V., Corneil D., Brea J. (2018). Eligibility traces and plasticity on behavioral time scales: experimental support of neohebbian three-factor learning rules. Front. Neural Circ..

[54] Hubara I., Courbariaux M., Soudry D., El-Yaniv R., Bengio Y. (2016).

[55] Tokui S., Sato I. (2017). International Conference on Machine Learning.

[56] Hou L., Yao Q., Kwok J.T. (2017). International Conference on Learning Representations.

[57] Yin P., Lyu J., Zhang S., Osher S., Qi Y., Xin J. (2019). International Conference on Learning Representations.

[58] Krizhevsky A., Hinton G. (2009).

[59] Li H., Liu H., Ji X., Li G., Shi L. (2017). Cifar10-dvs: an event-stream dataset for object classification. Front. Neurosci..

[60] Amir A., Taba B., Berg D., Melano T., McKinstry J., Di Nolfo C., Nayak T., Andreopoulos A., Garreau G., Mendoza M. (2017). The IEEE/CVF Computer Vision and Pattern Recognition Conference.

[61] Basalyga G., Salinas E. (2006). When response variability increases neural network robustness to synaptic noise. Neural Comput..

[62] McDonnell M.D., Ward L.M. (2011). The benefits of noise in neural systems: bridging theory and experiment. Nat. Rev. Neurosci..

[63] Gu F., Sng W., Hu X., Yu F. (2021). Eventdrop: Data augmentation for event-based learning. arXiv.

[64] Mainen Z.F., Sejnowski T.J. (1995). Reliability of spike timing in neocortical neurons. Science.

[65] Tiesinga P., Fellous J.-M., Sejnowski T.J. (2008). Regulation of spike timing in visual cortical circuits. Nat. Rev. Neurosci..

[66] de Ruyter van Steveninck R.R., Lewen G.D., Strong S.P., Koberle R., Bialek W. (1997). Reproducibility and variability in neural spike trains. Science.

[67] Rolls E.T., Deco G. (2010).

[68] Onken A., Liu J.K., Karunasekara P.P.C.R., Delis I., Gollisch T., Panzeri S. (2016). Using matrix and tensor factorizations for the single-trial analysis of population spike trains. PLoS Comput. Biol..

[69] McIntosh L., Maheswaranathan N., Nayebi A., Ganguli S., Baccus S. (2016). Advances in Neural Information Processing Systems.

[70] Zheng Y., Jia S., Yu Z., Liu J.K., Huang T. (2021). Unraveling neural coding of dynamic natural visual scenes via convolutional recurrent neural networks. Patterns.

[71] Yamins D.L.K., Hong H., Cadieu C.F., Solomon E.A., Seibert D., DiCarlo J.J. (2014). Performance-optimized hierarchical models predict neural responses in higher visual cortex. Proc. Natl. Acad. Sci. USA.

[72] Gerstner W., Kistler W.M. (2002).

[73] Vaswani A., Shazeer N., Parmar N., Uszkoreit J., Jones L., Gomez A.N., Kaiser Ł., Polosukhin I. (2017). Advances in Neural Information Processing Systems (NeurIPS).

[74] Zhang J., Dong B., Zhang H., Ding J., Heide F., Yin B., Yang X. (2022). The IEEE/CVF Computer Vision and Pattern Recognition Conference.

[75] Indiveri G., Liu S.-C. (2015). Memory and information processing in neuromorphic systems. Proc. IEEE.

[76] Pei J., Deng L., Song S., Zhao M., Zhang Y., Wu S., Wang G., Zou Z., Wu Z., He W. (2019). Towards artificial general intelligence with hybrid tianjic chip architecture. Nature.

[77] Davies M., Wild A., Orchard G., Sandamirskaya Y., Guerra G.A.F., Joshi P., Plank P., Risbud S.R. (2021). Advancing neuromorphic computing with loihi: A survey of results and outlook. Proc. IEEE.

[78] Benjamin B.V., Gao P., McQuinn E., Choudhary S., Chandrasekaran A.R., Bussat J.-M., Alvarez-Icaza R., Arthur J.V., Merolla P.A., Boahen K. (2014). Neurogrid: A mixed-analog-digital multichip system for large-scale neural simulations. Proc. IEEE.

[79] DeBole M.V., Taba B., Amir A., Akopyan F., Andreopoulos A., Risk W.P., Kusnitz J., Ortega Otero C., Nayak T.K., Appuswamy R. (2019). Truenorth: Accelerating from zero to 64 million neurons in 10 years. Computer.

[80] Davies M., Srinivasa N., Lin T.-H., Chinya G., Cao Y., Choday S.H., Dimou G., Joshi P., Imam N., Jain S. (2018). Loihi: A neuromorphic manycore processor with on-chip learning. IEEE Micro.

[81] Qiao N., Mostafa H., Corradi F., Osswald M., Stefanini F., Sumislawska D., Indiveri G. (2015). A reconfigurable on-line learning spiking neuromorphic processor comprising 256 neurons and 128k synapses. Front. Neurosci..

[82] Hazan A., Ezra Tsur E. (2022). Neuromorphic neural engineering framework-inspired online continuous learning with analog circuitry. Appl. Sci..

[83] White J.A., Rubinstein J.T., Kay A.R. (2000). Channel noise in neurons. Trends Neurosci..

[84] Grossman S., Yeagle E.M., Harel M., Espinal E., Harpaz R., Noy N., Mégevand P., Groppe D.M., Mehta A.D., Malach R. (2019). The noisy brain: power of resting-state fluctuations predicts individual recognition performance. Cell Rep..

[85] Masland R.H. (2004). Neuronal cell types. Curr. Biol..

[86] Klindt D., Ecker A.S., Euler T., Bethge M. (2017). Neural system identification for large populations separating “what” and “where”. Adv. Neural Inf. Process. Syst..

[87] Zhuang C., Yan S., Nayebi A., Schrimpf M., Frank M.C., DiCarlo J.J., Yamins D.L.K. (2021). Unsupervised neural network models of the ventral visual stream. Proc. Natl. Acad. Sci. USA.

[88] Cadena S.A., Denfield G.H., Walker E.Y., Gatys L.A., Tolias A.S., Bethge M., Ecker A.S. (2019). Deep convolutional models improve predictions of macaque v1 responses to natural images. PLoS Comput. Biol..

[89] Ratan Murty N.A., Bashivan P., Abate A., DiCarlo J.J., Kanwisher N. (2021). Computational models of category-selective brain regions enable high-throughput tests of selectivity. Nat. Commun..

[90] Ma G. (2023).

[91] Tal D., Schwartz E.L. (1997). Computing with the leaky integrate-and-fire neuron: logarithmic computation and multiplication. Neural Comput..

[92] Brunel N., van Rossum M.C.W. (2007). Lapicque’s 1907 paper: from frogs to integrate-and-fire. Biol. Cybern..

[93] Xiao M., Meng Q., Zhang Z., He D., Lin Z. (2022). Advances in Neural Information Processing Systems.

[94] Van Kampen N.G. (1992).

[95] Kloeden P.E., Platen E. (1992). *Numerical solution of stochastic differential equations*.

[96] Barndorff-Nielsen O.E., Shephard N. (2001). Non-gaussian ornstein-uhlenbeck-based models and some of their uses in financial economics. J. Roy. Stat. Soc. B.

[97] Patel A., Kosko B. (2008). Stochastic resonance in continuous and spiking neuron models with levy noise. IEEE Trans. Neural Network..

[98] Plesser H.E., Gerstner W. (2000). Escape rate models for noisy integrate-and-free neurons. Neurocomputing.

[99] Jolivet R., Rauch A., Lüscher H.R., Gerstner W. (2006). Predicting spike timing of neocortical pyramidal neurons by simple threshold models. J. Comput. Neurosci..

[100] Burt J.M., Garman M.B. (1971). Conditional monte carlo: A simulation technique for stochastic network analysis. Manag. Sci..

[101] Titsias M.K., Lázaro-Gredilla M. (2015). Advances in Neural Information Processing Systems.

[102] Fiete I.R., Seung H.S. (2006). Gradient learning in spiking neural networks by dynamic perturbation of conductances. Phys. Rev. Lett..

[103] Shekhovtsov A., Yanush V., Flach B. (2020). Advances in Neural Information Processing Systems.

[104] Shrestha S.B., Orchard G.S. (2018). Advances in Neural Information Processing Systems.

[105] Mao X. (2007).

[106] Cubuk E.D., Zoph B., Mane D., Vasudevan V., Le Q.V. (2018). The IEEE/CVF Computer Vision and Pattern Recognition Conference.

[107] Samadzadeh A., Far F.S.T., Javadi A., Nickabadi A., Chehreghani M.H. (2020). Convolutional spiking neural networks for spatio-temporal feature extraction. arXiv.

[108] Kingma D.P., Ba J. (2014). Adam: A method for stochastic optimization. arXiv.

[109] Loshchilov I., Hutter F. (2016). Sgdr: Stochastic gradient descent with warm restarts. arXiv.

[110] Zheng H., Wu Y., Deng L., Hu Y., Li G. (2021). Going deeper with directly-trained larger spiking neural networks. Proc. AAAI Conf. Artif. Intell..

[111] Gu P., Xiao R., Pan G., Tang H.S. (2019). International Joint Conferences on Artifical Intelligence.

[112] Wu Z., Zhang H., Lin Y., Li G., Wang M., Tang Y. (2022). Liaf-net: Leaky integrate and analog fire network for lightweight and efficient spatiotemporal information processing. IEEE Transact. Neural Networks Learn. Syst..

[113] Liu J., Yang X., Zhu Y., Lei Y., Cai J., Wang M., Huan Z., Lin X. (2021). How neuronal noises influence the spiking neural networks’s cognitive learning process: A preliminary study. Brain Sci..

[114] Lezcano-Casado M. (2019). Advances in Neural Information Processing Systems.

[115] Goodfellow I.J., Shlens J., Szegedy C. (2015). International Conference on Learning Representations.

[116] Fano U. (1947). Ionization yield of radiations. ii. the fluctuations of the number of ions. Phys. Rev..

[117] Park I.M., Seth S., Paiva A.R., Li L., Principe J.C. (2013). Kernel methods on spike train space for neuroscience: a tutorial. IEEE Signal Process. Mag..

[118] Arribas D., Zhao Y., Park I.M. (2020). Rescuing neural spike train models from bad mle. Adv. Neural Inf. Process. Syst..

[119] Zenke F., Ganguli S. (2018). Superspike: Supervised learning in multilayer spiking neural networks. Neural Comput..

